# Presaccadic attention does not facilitate the detection of changes in the visual field

**DOI:** 10.1371/journal.pbio.3002485

**Published:** 2024-01-25

**Authors:** Priyanka Gupta, Devarajan Sridharan

**Affiliations:** Centre for Neuroscience, Indian Institute of Science, Bangalore, India; Yeshiva University Albert Einstein College of Medicine, UNITED STATES

## Abstract

Planning a rapid eye movement (saccade) changes how we perceive our visual world. Even before we move the eyes visual discrimination sensitivity improves at the impending target of eye movements, a phenomenon termed “presaccadic attention.” Yet, it is unknown if such presaccadic selection merely affects perceptual sensitivity, or also affects downstream decisional processes, such as choice bias. We report a surprising lack of presaccadic perceptual benefits in a common, everyday setting—detection of changes in the visual field. Despite the lack of sensitivity benefits, choice bias for reporting changes increased reliably for the saccade target. With independent follow-up experiments, we show that presaccadic change detection is rendered more challenging because percepts at the saccade target location are biased toward, and more precise for, only the most recent of two successive stimuli. With a Bayesian model, we show how such perceptual and choice biases are crucial to explain the effects of saccade plans on change detection performance. In sum, visual change detection sensitivity does not improve presaccadically, a result that is readily explained by teasing apart distinct components of presaccadic selection. The findings may have critical implications for real-world scenarios, like driving, that require rapid gaze shifts in dynamically changing environments.

## Abstract 2

Our perception of an object sharpens momentarily, even as we plan to gaze towards it. This study shows that this fails to happen when the object changes subtly, suggesting that gaze plans may affect visual abilities in surprising ways in changing environments.

## Introduction

Saccades are ballistic eye movements that enable us to bring into focus important parts of the visual scene, in quick succession, for foveal analysis. But even before the eyes move visual sensitivity improves at the location of the impending saccade: a phenomenon termed “presaccadic attention.”

An extensive literature has characterized the behavioral benefits of presaccadic attention. Both visual discrimination and identification accuracy have been reported to improve presaccadically [1–5]. In particular, many studies have reported presaccadic improvements in orientation discrimination accuracy [6–12]. Several mechanisms have been proposed to explain these effects. Early work on the premotor theory of attention suggested that attentional shifts were tightly linked to the activation of oculomotor circuitry [13,14]. More recent behavioral evidence suggests that presaccadic attention narrows orientation tuning [15–17] and preferentially enhances high spatial frequency information [15,18,19]. These effects, in conjunction with a perisaccadic compression of visual space [20–22], may lead to higher discrimination accuracies for oriented gratings at the saccade target location. Moreover, orientation information at the saccade target biases processing across the visual field [23,24] (but see [25,26]), representing a selection bias for saccade-congruent features. Furthermore, saccade preparation increases the perceived contrast at the saccade target location [6], and produces response gain-like effects [10], suggesting that encoding strength increases presaccadically. While the spatial spread of presaccadic attention is strongly determined by visual context [27–30], a decrement in sensitivity occurs at non-saccade target locations sufficiently far removed (e.g., different hemifields) from the saccade target, suggesting a capacity-limited presaccadic selection mechanism [8,31].

Despite this significant literature characterizing the effects of presaccadic attention, it is unclear how presaccadic selection mechanisms influence perceptual versus decisional processing [8,15]. The vast majority of previous literature relied on discrimination tasks employing conventional forced choice responses [5,6,9,12,27,32], which isolate the effects of presaccadic selection on perceptual sensitivity by controlling for choice biases. But, such visual discrimination tasks, by design, do not permit quantifying the effect of presaccadic selection mechanisms on choice biases that operate across visual space [33–37].

Here, we employ signal detection theory [33,34] and Bayesian modeling of behavior to distinguish perceptual and decisional mechanisms underlying presaccadic visual change detection. We document a surprising absence of presaccadic perceptual benefits in visual change detection tasks. With a novel change detection paradigm and multidimensional signal detection theory, we quantified distinct components of presaccadic selection—sensitivity and criteria—at the saccade target and non-saccade target locations. Surprisingly, change detection sensitivity was not enhanced presaccadically, at the saccade target location. Rather, choice criterion was the lowest, and spatial choice bias highest, for reporting a change at the saccade target. To further investigate these findings, we tested presaccadic effects on orientation estimation with 2 stimuli, presented sequentially. Orientation estimates were more precise at the saccade target location, but only for the most recent of the 2 stimuli. Additionally, the more recent stimulus biased perceptual orientation estimates of the previous stimulus at the saccade target location. Analysis with a “variable precision” model [38,39] revealed that incorporating both perceptual and choice biases was essential to fully account for the behavioral consequences of presaccadic selection.

In conjunction with Bayesian modeling of behavior, the results identify the distinct contributions of perceptual and decisional processes to the behavioral effects of presaccadic selection. Our finding of a robust “presaccadic recency bias” has critical implications for real-world scenarios (e.g., driving, sports) in which frequent gaze shifts occur in dynamically changing environments.

## Results

### Spatial choice bias but not sensitivity is enhanced presaccadically

**Fig 1:**
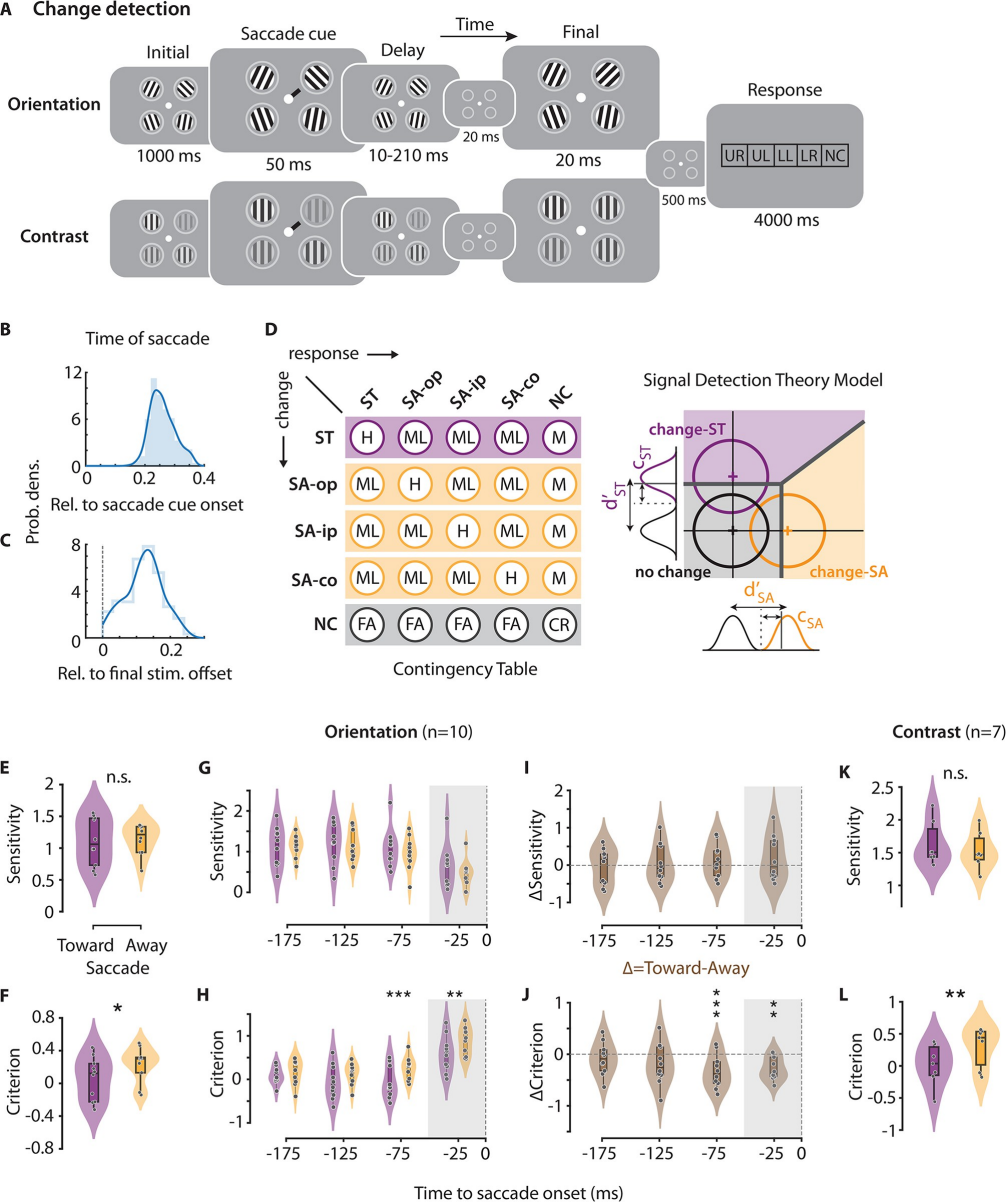
Effects of presaccadic selection on visual change detection sensitivity and criterion. **(A)** Dual task paradigm for quantifying presaccadic change detection performance in multialternative orientation change detection (top row) and contrast change detection (bottom row) tasks. Participants detected and localized changes in Gabor orientation (top) or contrast (bottom) while also planning a saccade to the cued location (see text for details). Rightmost panel: Response box configuration. UR, upper right; UL, upper left; LL, lower left; LR, lower right; NC, no change. **(B)** Distribution (filled histogram) of the interval between saccade cue onset and saccade onset, across trials, for a representative (median) participant. Fit: kernel density estimate. **(C)** Distribution (open histogram) of the interval between the final stimulus offset and saccade onset, for the same participant as in panel B. Other conventions are the same as in panel B. **(D)** (Left) Stimulus-response contingency table for a multialternative change detection and localization (4-ADC) task. Rows: stimulus events, columns: response types. H, hits; M, misses; ML, mislocalizations; FA, false alarms; CR, correct rejections (see Methods for more details). ST, Saccade Toward; SA, Saccade Away; ip, ipsilateral; op, opposite; co, contralateral (all relative to the Saccade Toward location); NC, no change**.** (Right) Multidimensional signal detection model for estimating criterion and sensitivity at each location (see Methods for details). Change-ST: change at saccade target location (purple distribution and decision zone); Change-SA: change at saccade away location (orange distribution and decision zone); No change: no change at either location (black/gray distribution and decision zone). **(E)** Sensitivity for orientation change detection reports at the saccade target (Toward) and non-saccade target (Away) locations (*n* = 10 participants). Violin: rotated kernel density estimates; center line: median; box limits: upper and lower quartiles; whiskers: 1.5× interquartile range. All data points are shown. Purple: Saccade Toward (ST) location. Orange: Saccade Away (SA) location. **(F)** Same as in panel E, but showing criterion for orientation change detection and localization. **(G)** Same as in panel E, but showing the time course of sensitivity at ST and SA locations, locked to the saccade onset (dashed vertical line). Shaded gray zone: saccadic suppression epoch. **(H)** Same as in panel G, but showing the time course of criterion at ST and SA locations, locked to the saccade onset. **(I)** Time course of sensitivity modulation—difference in sensitivity at the Saccade Toward and Away locations—locked to saccade onset (dashed vertical line). Δ = Toward–Away. **(J)** Same as in panel I, but showing time course of criterion modulation—difference in criterion at the Saccade Toward and Away locations—locked to saccade onset. **(H–J)** Other conventions are the same as in panel G. **(K)** Same as in panel E, but showing sensitivity at the Saccade Toward and Away locations (*n* = 7 participants) for the contrast change detection task. **(L)** Same as in panel F, but showing criterion for contrast change detection task. All panels. Asterisks: significance levels (* *p* < 0.05, ** *p* < 0.01, *** *p* <0.001). n.s., not significant. **(F–L)** Other conventions are the same as in panel E. Data are available at https://dx.doi.org/10.6084/m9.figshare.21792002.

To explore the effects of presaccadic selection on visual change detection, we developed a dual task paradigm that enabled quantifying change detection sensitivity and criterion at multiple locations on the screen as participants prepared and executed saccades. Briefly, on each trial, participants (*n* = 10) were presented with 4 oriented Gabor patches, 1 in each visual quadrant, and cued to make a saccade to one of the 4 stimuli (25% probability across locations) as quickly and accurately as possible (Fig 1A–1C). A short, variable interval after the cue—but before the saccade was initiated—the stimuli were blanked briefly (20 ms). Following their reappearance any one (or none) of the Gabor stimuli had changed in orientation (Fig 1A, top). Participants detected and localized the change with a 5-alternative response (Fig 1A). Sensitivity and criterion were quantified from behavioral responses (Fig 1D, left) with a well-validated signal detection model [33,34] (Methods; Fig 1D, right). The location cued for saccades was sampled independently from and was, therefore, not predictive of the location of change. This design enabled us to quantify the effects of presaccadic selection, without the confounding effects of voluntary (endogenous) spatial attention, on psychophysical parameters (sensitivity and criterion). Sensitivity and criterion were quantified at the saccade target location (ST or “toward”) and compared with their average value at the 3 other locations away from the saccade target (SA or “away”).

Given the extensive literature on presaccadic attention, we expected to find significant behavioral facilitation at the saccade target location. Surprisingly, change detection sensitivity was not higher at the saccade target (Saccade Toward/ST) location than at the other (Saccade Away/SA) locations (Fig 1E) (d’_Toward_ = 1.1 ± 0.12, d’_Away_ = 1.15 ± 0.08, *p* = 0.655; random permutation test; psychophysical parameters averaged across the 3 away locations). On the other hand, change detection criteria were lower—and spatial choice bias higher—at the saccade target location, relative to the other locations (Fig 1F; c_Toward_ = 0.06 ± 0.08, c_Away_ = 0.24 ± 0.07, *p* = 0.013). Thus, presaccadic selection yielded a near-optimal choice criterion (approximately 0) at the ST location, and a more conservative criterion at the other (SA) locations suggesting a higher spatial choice bias for change detection at the ST location.

Because presaccadic effects are known to be transient [7,11], we tested if more robust sensitivity (or criterion) modulations occurred in time windows proximal to the saccade. For this, we analyzed the dynamics of sensitivity and criterion changes in 4 non-overlapping time windows (interval: –200 ms to 0 ms; duration: 50 ms) locked to saccade onset. The variable interval between the saccade cue and the change event—as well as the natural variability in saccade onset times following cue presentation—enabled us to quantify sensitivity and criteria at different latencies of the change event relative to saccade onset. Sensitivity was not different between the ST and SA locations in any time window tested (*p* > 0.05 for all windows tested) (Fig 1G and 1I). On the other hand, criterion modulation was strong and significant in the 0 to 50 ms (*p* = 0.006) and 50 to 100 ms (*p* < 0.001) presaccadic windows, trending toward significance in a 100 to 150 ms window (*p* = 0.062) but not significant in a 150 to 200 ms window (*p* = 0.205) (Fig 1H and 1J). While the earliest of these windows (0 to 50 ms) is likely subject to saccadic suppression (Fig 1G–1J, gray shading), the penultimate window (50 to 100 ms) likely characterizes presaccadic selection (see Discussion). These results were confirmed with a finer-grained temporal analysis of raw behavioral responses—sum of hit and false alarm rates—that govern criterion estimates (S1A and S1B Fig).

We performed additional control investigations to assess the validity and generality of these results. First, we tested whether the execution of the saccade itself was biased by the occurrence of the change either at the ST location or the SA locations. The median saccade latencies were not significantly different between the no-change trials (259 ± 7 ms) and the trials in which a change occurred (260 ± 7 ms) (*p* > 0.9, Mann–Whitney U test). Saccade latencies were also comparable across trials in which a change occurred at the ST location (262 ± 8 ms) versus at one of the SA locations (260 ± 7 ms; *p* > 0.9). Similarly, the dispersion of the saccade endpoints around the center of the saccade target stimulus was not significantly different across the trials with change at the ST location (mean deviation: 1.32 ± 0.10 dva), change at any SA location (1.32 ± 0.08 dva), and no-change trials (1.30 ± 0.09 dva) (*p* > 0.05, pairwise signed rank test). Together, these suggest that the saccade execution itself was not affected by the spatial location of change occurrence.

Second, we tested whether perisaccadic mislocalization [20,22] could have resulted in a lower criterion at the ST location. For this, we quantified and compared the proportion of change mislocalizations toward the ST location when a change occurred at any of SA locations to the proportion of change mislocalizations toward the SA locations when a change occurred at the ST location. If perisaccadic compression of visual space toward the ST location contributed to spatial mislocalization biases, this would predict a higher proportion of ST mislocalizations for changes occurring at SA locations, as compared to the converse. On the other hand, we observed that mislocalization rate toward the ST location when a change occurred at any other SA location (0.10 ± 0.01) was not significantly different from the mislocalization rate toward SA locations when a change occurred at the ST location (0.13 ± 0.02) (*p* > 0.05, permutation test). In other words, changes that occurred away from the saccade target were no more likely to be reported at the saccade target location than vice versa. This suggests that the observed modulation in criterion was not a direct consequence of peri-saccadic mislocalization. We further confirmed this result by entirely removing all trials corresponding mislocalization responses from the stimulus-response contingency table (Fig 1D). The remaining observations—hits, misses, false alarms, and correct rejections—were then analyzed with a one-dimensional signal detection theory model (Methods). Again, we observed a significant presaccadic difference between the ST and SA locations in terms of criterion (*p* = 0.026), but not sensitivity (*p* = 0.379), modulation.

Third, strong sensory transients during the presaccadic epoch can delay saccades by resetting the saccade program; this effect is particularly strong for transients that occur early on in the presaccadic epoch [40–43]. We tested for saccadic inhibition by analyzing whether the distribution of saccade onset times aligned to blank onset (“transient”-locked saccade onsets) displayed bimodality; a dip in saccade likelihood approximately 50 to 70 ms after the transient would suggest saccade resetting [42]. But we observed no strong evidence for such bimodality, in either the combined data (*p* = 0.982, Hartigan’s dip test [44]), or for any of the participants, individually (all *p* > 0.05, median *p* = 0.983) (S2 Fig). Nonetheless, it is possible that saccades were inhibited on a subset of trials, or to a lesser extent than with other task paradigms. To address this possibility, we reanalyzed the trials in which the change onset occurred <70 ms before saccade onset, i.e., in time windows likely to escape saccade resetting. Even upon restricting our analyses to these trials, we observed a significant modulation of criterion (*p* < 0.001), but no modulation of sensitivity (*p* = 0.274) (S1C–S1E Fig).

Fourth, we tested for differences in sensitivity or criterion effects across the 3 non-saccade target locations. Detection sensitivities were not significantly different across the SA locations (*H*(2) = 0.64, *p* = 0.727, Kruskal–Wallis test) sensitivity modulation—the difference in sensitivity between the ST and the respective SA location—was also not significantly different across the locations (*H*(2) = 0.65, *p* = 0.722). Similarly, criteria were not significantly different across the SA locations (*H*(2) = 1.64, *p* = 0.440), nor was criterion modulation significantly different across these locations (*H*(2) = 0.58, *p* = 0.750).

Fifth, we tested if sensitivity effects would occur for other change angles. Because in the main experiment, we had tested a single orientation change angle—staircased independently for each participant (Methods)—we repeated this experiment with *n* = 5 participants, a subset of main cohort, by measuring the entire psychophysical function across multiple orientation change angles (Methods). Again, we observed that detection sensitivity was not significantly different between the saccade target location and the other locations for any of the angles tested (*p* = 0.216) (S1F Fig).

Finally, we evaluated the generality of these results in another dual task paradigm involving contrast step detection performed by 7 participants, including 4 from the earlier cohort. The task design was identical to that of the main experiment, except that participants had to detect and localize a contrast increment—rather than an orientation change—that occurred in one of the 4 Gabor patches (Fig 1A, bottom, Methods). Consistent with the main experiment findings, detection sensitivity was not significantly different across locations (Fig 1K) (d’_Toward_ = 1.63 ± 0.13, d’_Away_ = 1.53 ± 0.11, *p* = 0.177, random permutation test), but detection criterion was lowest at the saccade target location (Fig 1L) (c_Toward_ = 0.01 ± 0.12, c_Away_ = 0.31 ± 0.12, *p* = 0.004). Again, sensitivity was not different between the ST and SA locations in any time window tested (*p* > 0.05 for all windows tested) (S1G Fig). But, strongest criterion modulations occurred 50 to 100 ms prior to the saccade (*p* < 0.001) (S1H Fig).

To further confirm the robustness of these findings, we performed Bayesian sequential analysis to check how evidence for the null versus the alternative hypotheses changed as data from successive participants were acquired (S3A and S3B Fig; see also Methods). Across all data points acquired, we estimated a Bayes factor (BF; Methods) of 10.18 for criterion effects and a BF of 0.32 for sensitivity effects (data pooled across orientation change and contrast change detection tasks). These results provide strong evidence favoring a lower criterion at the saccade target location and substantial evidence against a difference in sensitivity across all locations.

In summary, no spatially specific improvement in change detection sensitivity occurred, presaccadically. Rather, change detection criteria were lowest, and spatial choice bias highest, at the saccade target; these effects were strongest at times closest to saccade onset. In other words, presaccadic selection induced a bias for detection choices selectively at the saccade target location, with no concomitant improvement in the ability to detect changes at this location.

### A presaccadic recency bias renders change detection challenging

**Fig 2:**
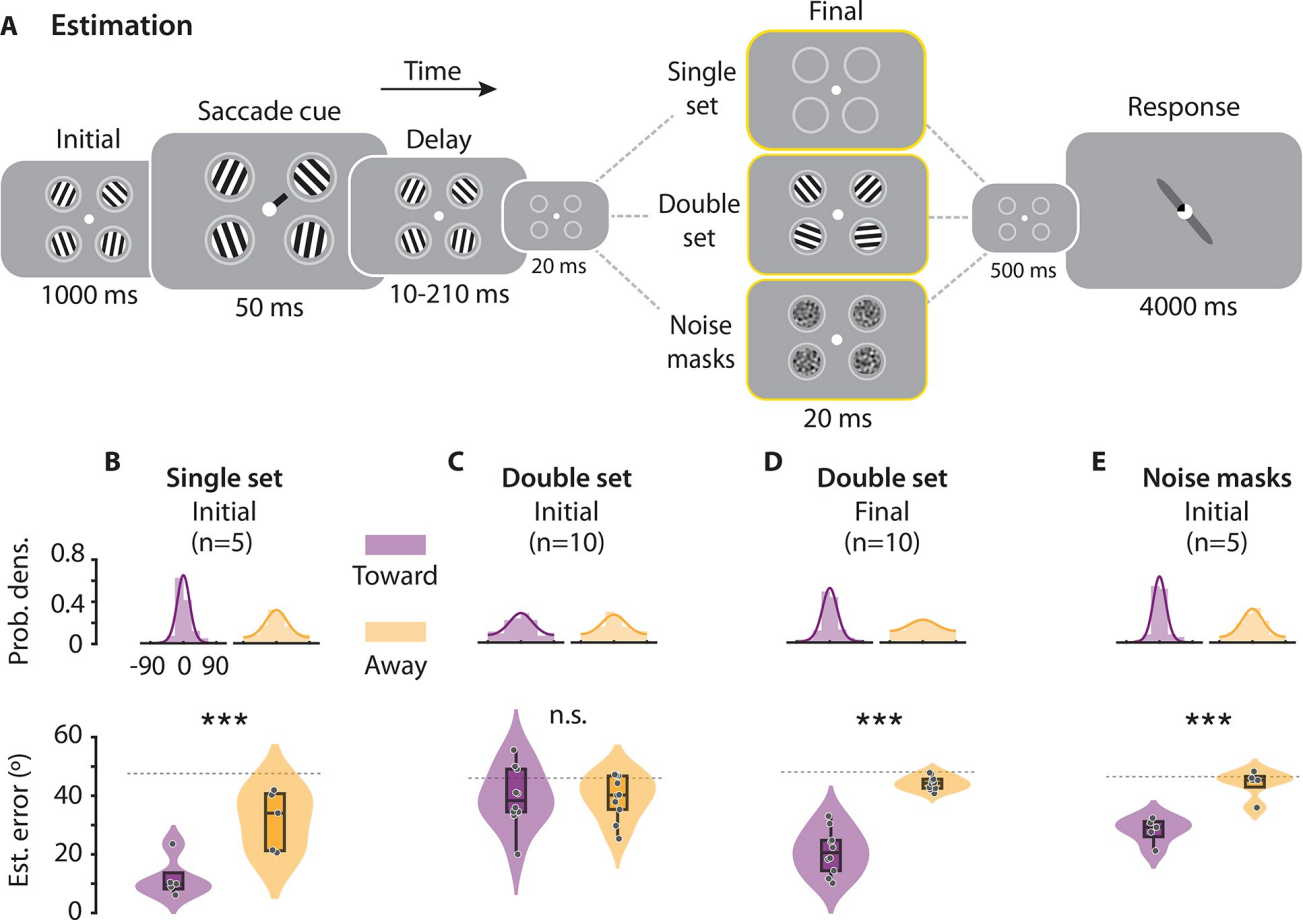
Effects of presaccadic selection on precision of orientation estimates. ** (A)** Dual task paradigm for presaccadic orientation estimation, with 3 different trial types: (yellow outline, upper row) single set trials, (yellow outline, middle row) double set trials, and (yellow outline, lower row) noise mask trials. Participants estimated the orientation of either the initial or the final set of Gabor stimuli in distinct blocks of trials, while also planning a saccade toward the cued location (see text for details). **(B)** (Top) Distribution of estimation errors at the Saccade Toward (left, purple) and Away (right, orange) locations for single set trials. Lines: von Mises fits. (Bottom) MAE of orientation estimates at the ST (left) and SA (right) locations, for the initial stimulus of single set trials (*n* = 5 participants). **(C)** Same as in panel B, but showing error distributions and MAE for the initial stimulus of double set trials. **(D)** Same as in panel B, but showing error distributions and MAE for the final stimulus of double set trials. **(E)** Same as in panel B, but showing error distributions and MAE for the initial stimulus of noise mask trials. **(B–E)** Other conventions are the same as in Fig 1E. Data are available at https://dx.doi.org/10.6084/m9.figshare.21792002. MAE, Mean absolute error; SA, Saccade Away; ST, Saccade Toward.

To further understand this surprising lack of presaccadic change detection benefits, we conducted another experiment involving presaccadic orientation estimation. The stimuli and saccade cue timings were identical in nearly all respects to the previous dual task—with 2 sets of 4 Gabors presented sequentially—except that participants (*n* = 10) reported the orientation of one of the 8 Gabors that was probed post hoc (Fig 2A). In distinct blocks of trials, one of the 4 stimuli in either the initial set or the final set were probed exclusively (“double set” trials, Fig 2A, yellow outline, middle row); participants were informed beforehand regarding which set—initial or final—would be probed for that entire block (Methods). Participants reported the orientation of the probed Gabor by rotating a response bar (Fig 2A, rightmost). In a subset of randomly interleaved trials (40%), the final Gabor stimulus set was replaced with either a blank (*n* = 5 participants) or with spatially filtered noise masks (*n* = 5 participants). Thus, in the former trial type only 1 set of Gabors was presented (“single set” trials; Fig 2A, yellow outline, uppermost), whereas in the latter trial type, the initial Gabor stimulus set was followed by a blank and noise masks (“noise mask” trials; Fig 2A, yellow outline, lowermost).

Single set trials revealed systematic benefits of presaccadic attention: Orientation estimation accuracy was significantly higher, and absolute error of orientation estimates (“estimation error”) nearly 2× lower, at the saccade target location than at the other, non-saccade target, locations (Fig 2B) (mean ± s.e.; Err_Toward_ = 14.4° ± 2.6°, Err_Away_ = 31.5° ± 3.9°, *p* < 0.001, permutation test; BF = 22.51); similar results were obtained with quantifying the precision (Methods) of orientation estimates (Fig 2B, inset; Precision_Toward_ = 1.30 ± 0.39, Precision_Away_ = 0.66 ± 0.08; *p* < 0.001, random permutation test). In other words, when only one stimulus was presented at the saccade target location, presaccadic attention improved the precision of orientation estimates, in line with results reported in multiple previous studies [6,8,11,16,17].

Yet, in double set trials—when the initial set of stimuli was followed by a second (final) set—this presaccadic benefit vanished entirely for the initial Gabor stimulus. Estimation error for the initial Gabor stimulus was not different between the saccade target location and the other locations (Fig 2C) (Err_Toward_ = 39.5° ± 2.8°, Err_Away_ = 39.5° ± 2.0°, *p* = 0.526, random permutation test; BF = 0.3). Remarkably, a significant presaccadic benefit occurred, now, for the most recent Gabor stimulus: estimation error for the final Gabor stimulus was lowest at the saccade target location, as compared to the other locations (Fig 2D) (Err_Toward_ = 21.7° ± 2.1°, Err_Away_ = 41.9° ± 0.6°, *p* < 0.001; BF > 10^3^). In other words, when 2 sets of stimuli were presented in sequence, presaccadic benefits occurred only for the last of the 2 stimuli.

We tested whether the loss of precision could be accounted for by visual “masking” of the initial stimulus by the final stimulus. For this, we presented spatially filtered noise masks, rather than Gabor stimuli, in the final set (Fig 2A, lowermost, Methods). Interestingly, in this case, presaccadic benefits persisted at the saccade target location (Err_Toward_ = 29.3° ± 1.7°, Err_Away_ = 43.0° ± 1.8°, *p* < 0.001, permutation test; BF = 32.98) (Fig 2E). In other words, sensory masking effects could not explain the loss in precision for the initial stimulus in the double set trials. Rather, the final stimulus must contain task relevant features—in this case, an oriented Gabor stimulus—to abolish presaccadic benefits for the initial stimulus.

**Fig 3:**
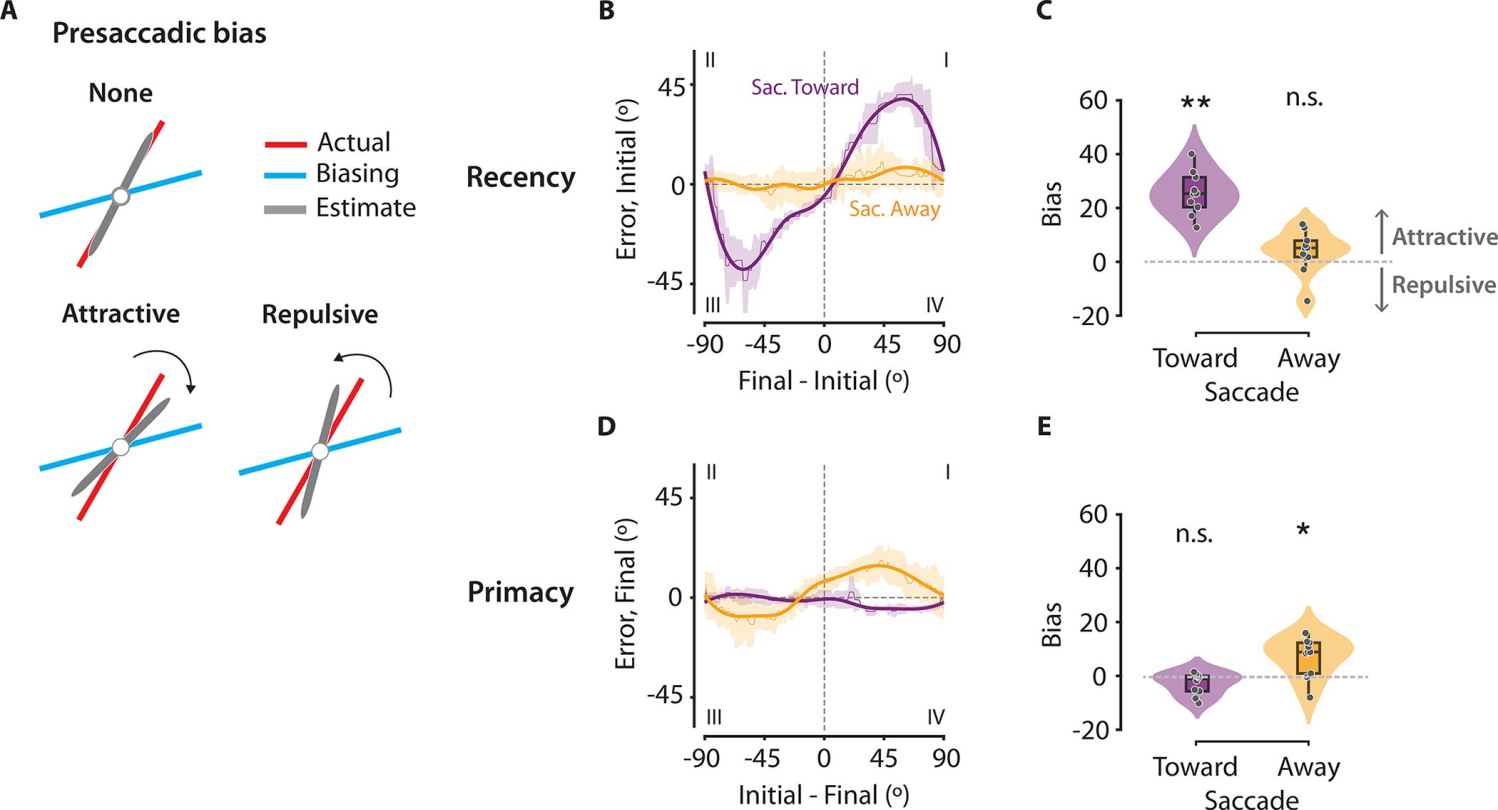
Evidence for a presaccadic recency bias in orientation estimation. **(A)** Schematic showing an attractive bias (bottom, left), a repulsive bias (bottom, right), and no bias (top). In the case of an attractive bias, orientation estimates (gray) of the actual stimulus (red) are biased toward the orientation of the biasing stimulus (blue). In the case of the repulsive bias, orientation estimates are biased away from the biasing stimulus. **(B, C)** Recency bias: the bias in the orientation estimate of the initial stimulus induced by the final stimulus. **(B)** Estimation error for the initial stimulus (y-axis) plotted against the difference between orientations of the final and initial stimuli (x-axis), at the ST (purple) and the SA (orange) locations. Dashed horizontal and vertical lines: zero estimation error and zero orientation difference, respectively. Thick, solid lines: Cubic spline fits. **(C)** Recency bias at the ST (left, purple) and SA (right, orange) locations in the double set trials, quantified using the signed area under the curve, from panel B (Methods). Positive and negative values denote an attractive or a repulsive bias, respectively. Other conventions are the same as in Fig 1E. **(D, E)** Primacy bias: the bias in the orientation estimate of the final stimulus induced by the initial stimulus. **(D)** Same as in panel B, but showing the estimation error for the final stimulus (y-axis) plotted against the difference between orientations of the initial and final stimuli (x-axis). Other conventions are the same as in panel B. **(E)** Same as in panel C, but showing primacy bias. Other conventions are the same as in panel C. Data are available at https://dx.doi.org/10.6084/m9.figshare.21792002. SA, Saccade Away; ST, Saccade Toward.

To investigate these temporal order effects further we tested whether, and how, the percept of the initial stimulus at the saccade target location was influenced by the final stimulus (Fig 3A). Indeed, orientation estimates of the initial stimulus were strongly and systematically biased toward the orientation of the final stimulus, even though the initial and final Gabor stimuli were uniformly and independently sampled (Fig 3B and 3C) (Bias = 25.7 ± 2.6 a.u., *p* = 0.002, Wilcoxon signed rank test; BF > 10^3^) (refer Methods for details regarding bias quantification). In contrast, the orientation estimates of the final stimulus were not biased by the orientation of the initial stimulus (Fig 3D and 3E) (Bias = −2.7 ± 1.3, *p* = 0.193; BF = 1.37). Interestingly, at the non-saccade target locations we observed the converse trend: orientation reports of the final stimulus were marginally biased toward the initial stimulus orientation (Bias = 7.4 ± 2.3, *p* = 0.014; BF = 5.68), but not vice versa (Bias = 4.0 ± 2.6, *p* = 0.131; BF = 0.76). A Bayesian sequential analysis robustness check is presented in S3C–S3F Fig.

We tested if these effects were not due to presaccadic temporal order reversal effects—an illusory reversal in sequence order of a sequence that occurs approximately 50 ms before a saccade [45,46]. We re-evaluated the presaccadic recency and primacy biases by excluding trials where the offset of the final set of stimuli was within 50 ms of the saccade onset (S4A and S4B Fig). The estimates of the initial stimulus continued to be biased towards the final stimulus at the saccade target location (Bias = 24.6 ± 2.7, *p* = 0.002), confirming that the presaccadic recency bias was not due to an apparent confusion in the stimulus order.

To summarize, the precision of orientation estimates was highest at the saccade target location, but only for the more recent of the 2 stimuli presented at this location. Moreover, orientation estimates for the initial stimulus were biased toward the orientation of the recent stimulus. This presaccadic “recency” bias, therefore, rendered accurate change detection at the saccade target location potentially even more challenging.

### Both perceptual and choice biases reflect presaccadic selection effects

Although the recency bias could explain the lack of presaccadic benefits on change detection sensitivity, it appears at odds with our finding regarding a higher spatial choice bias for reporting changes at the saccade target location. Because of the recency bias, the perceived difference in orientation between the initial and final Gabors would always be smaller in magnitude than the actual difference. This would yield fewer false alarms, a higher criterion, and, therefore, a lower bias at the saccade target location; whereas we observed the opposite trend—a lower criterion, and higher bias—at the saccade target location.

**Fig 4:**
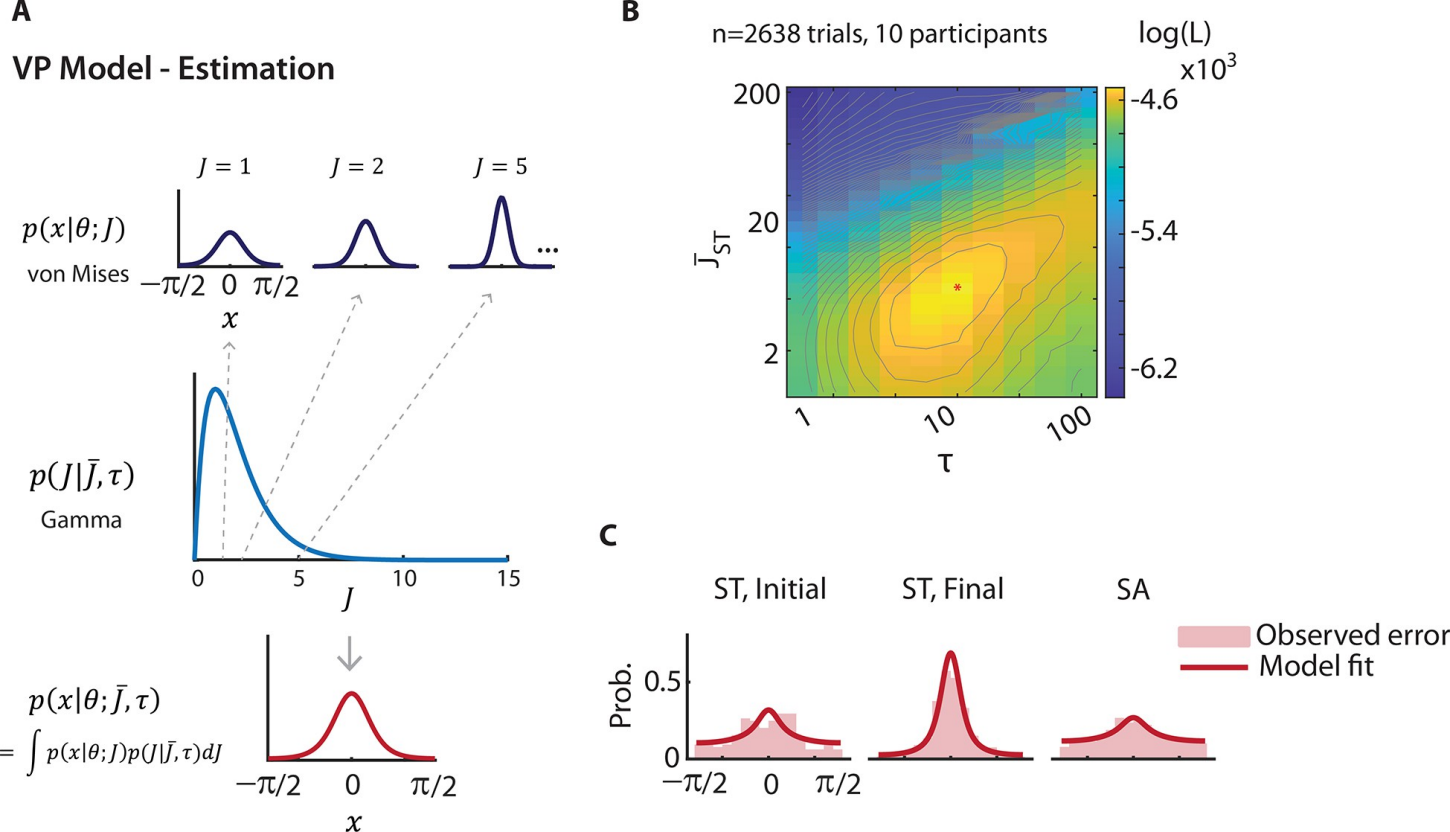
Variable precision model fits presaccadic selection’s effects on orientation estimation. **(A)** Schematic of the variable precision (VP) model for orientation estimation. (Top row) On each trial, internal measurement, x, of a stimulus with orientation, θ, follows a von Mises distribution, with a concentration parameter κ determined by precision parameter J. (Middle row) Across trials the precision is gamma distributed, with a mean $\bar{J}$ and scale parameter τ. (Bottom row) The distribution of the internal measurement x across trials is a mixture of von Mises distributions, marginalized across precision values. **(B)** Log likelihood (log (L)) landscape as a function of precision for the final stimulus at the ST location (${\bar{J}}_{ST}$) and scale, τ; although log likelihood is a function of all model parameters, these 2 parameters were chosen for illustrative purposes. Warmer colors indicate higher likelihoods. Red asterisk: Parameters corresponding to maximum log likelihood. Gray lines: contours of iso-log likelihood. **(C)** Left to right: Fits of the variable precision model (solid red lines) to the observed responses (light red histograms) for the initial ST, final ST and SA (pooled) stimuli, respectively, obtained with maximum likelihood estimation, based on the parameters in panel B. Data are available at https://dx.doi.org/10.6084/m9.figshare.21792002. SA, Saccade Away; ST, Saccade Toward.

We sought to reconcile these apparently contradictory findings using a Bayesian ideal observer model: the variable precision (VP) model [38,39]. The VP model has been successfully applied for modeling behavior in multialternative choice tasks [38,39]; in our study, it provided a unified framework for modeling behavior both in the orientation change detection task (Fig 1) and the orientation estimation task (Fig 2). Briefly, the model posits that neural resources, and, therefore, the precision of stimulus representation, are variable across items and trials. The model estimates 2 parameters, $\bar{J}$ and *τ* (scale), from which the mean and the variance of the stimulus precision can be computed (as $\bar{J}$ and $\bar{J}\tau$, respectively, Fig 4A) (Methods). Specifically, we tested whether one or both types of presaccadic bias—the perceptual (recency) bias, observed in the estimation task or the decisional (choice) bias, observed in the change detection task—would be relevant for explaining the effects of presaccadic selection.

First, to limit the number of parameters required to fit the orientation change detection task, we identified *τ* (scale) a priori by fitting the VP model to response error distributions from the orientation estimation task. Based on our empirical findings (Fig 2C and 2D), we employed 3 different mean precision parameters: 2 to model the different precisions for the initial and final stimuli at the saccade target location and 1 to model the precision for both stimuli at the non-saccade target locations. In line with experimental observations, the VP model estimated a significantly higher mean precision for the final ST stimulus, as compared to both the initial ST stimulus as well as both set of SA stimuli (Fig 4B) (${\bar{J}}_{ST,\mathrm{initial}}$ = 1.3, ${\bar{J}}_{ST,\mathrm{final}}$ = 6.4, ${\bar{J}}_{SA}$ = 1.0; *p* < 0.001, permutation test, data pooled across subjects). In addition, *τ* was estimated to be 13.0. Visualizing the fit of the VP model to the error distributions (Fig 4C), revealed that the model successfully captured the effect of presaccadic attention on the precision of orientation estimates (*p* > 0.1, Kuiper’s test).

**Fig 5:**
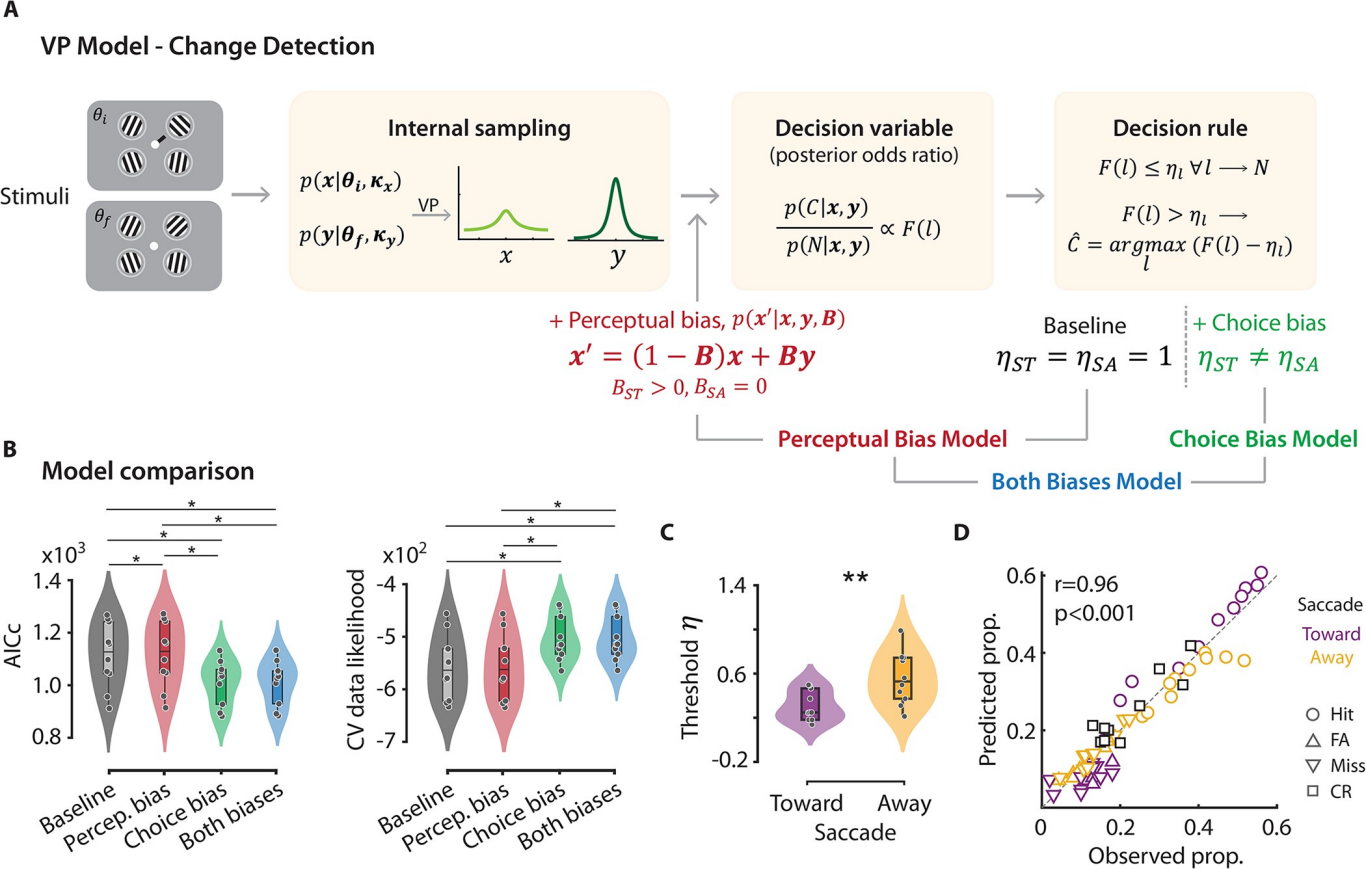
Perceptual and choice biases contribute to presaccadic selection’s effects on orientation change detection. **(A)** Schematic of the variable precision model for a change detection and localization task. (Left box) Internal measurements of the initial (**θ**_**i**_) and final (**θ**_**f**_) Gabor stimuli are represented by latent vectors (**x** and **y**, respectively) that follow von Mises distributions, with concentration parameters, **κ**. The encoding precision (**J**, directly related to **κ**) is variable across items and trials. (Middle box) F(l), the decision variable at each location, l, is proportion to the posterior odds ratio of probability of change (C) at that location to the probability of no change (N). The Bayesian ideal observer localizes the change to the location at which the decision variable F(l) exceeds the decision threshold η_l_ by the highest margin. If the decision variable does not exceed the decision threshold at any location, the observer reports “no change.” Perceptual bias model: The “baseline” VP model was modified to incorporate a “perceptual bias,” i.e., an attractive recency bias parameter, B, at the ST location (red text). Choice bias model: The “baseline” VP model was modified to incorporate unequal decision threshold parameters, η_l_, at the ST and SA locations (green text). Both biases model: Both perceptual bias and unequal decision threshold parameters were incorporated into the VP model (blue text). **(B)** (Left) Model comparison with the AICc, for the 4 VP models. Gray/black: baseline model, red: perceptual bias model, green: choice bias model, blue: both biases model. Box-and-whisker plot conventions are the same as in Fig 1E. (Right) Same as in the left panel, but showing the cross-validated data likelihoods for the 4 models. Other conventions are the same as in the left panel. **(C)** Same as in Fig 1E, but showing the decision threshold, η, estimated for the ST and SA locations with the “both biases” model. Other conventions are the same as in Fig 1E. **(D)** Response proportions predicted with the both biases model (y-axis) versus observed (true) response proportions (x-axis) in the change detection task. Circles: hits, upward triangles: FAs, downward triangles: misses, squares: correct rejections (CRs). Other conventions are the same as in panel C. Data are available at https://dx.doi.org/10.6084/m9.figshare.21792002. AICc, corrected Akaike information criterion; CR, correct rejection; CV, cross-validated; FA, false alarm; SA, Saccade Away; ST, Saccade Toward; VP, variable precision.

Next, we fit the VP model to the stimulus-response contingency table in the multialternative change detection task (Fig 5A) using a variant of the VP model [38]—a change localization model—that, on each trial, reports the location with the highest posterior probability of change occurrence. To model change detection, we modified the decision rule by incorporating a “decision threshold” for the posterior probability, below which the model provides a no-change response (Methods). We tested 3 variants of this model: (i) a “baseline” (standard VP) model that modeled unequal precisions at the ST and SA locations but imposed a common decision threshold of 1.0 across locations (Fig 5A, black); (ii) a “perceptual bias” model that extended the baseline model by additionally estimating a recency bias for the initial stimulus representation toward that of the final stimulus at the saccade target location (Fig 5A, red); and (iii) a “both biases” model that extended the perceptual bias model by additionally modeling choice bias, viz., different decision thresholds at each location (Fig 5A, blue). Models were formally compared with the corrected Akaike information criterion (AICc) and cross-validated data likelihoods.

In line with expectations the perceptual bias model did not improve baseline model fits (Fig 5B, left; median AICc_baseline_ = 1,128.5 ± 37.6; AICc_perceptualBias_ = 1,130.5 ± 37.7, *p* = 0.012). In fact, AICc values were marginally worse for the perceptual bias model: simply adding a recency bias at the ST location would yield lower (not higher) false alarms at this location, as noted above. In addition, evaluating the goodness-of-fit based on a randomization test (Methods) revealed successful fits for none of the 10 participants with either the baseline model or with the perceptual bias model. In stark contrast, upon incorporating differential decision thresholds at each location AICc values improved significantly (Fig 5B, left; AICc_bothBiases_ = 1,030.9 ± 26.0, *p* = 0.012). Moreover, the “both biases” model estimated a systematically lower decision threshold for the ST, as compared to the SA, location (Fig 5C, *η*_ST_ = 0.31 ± 0.04, *η*_SA_ = 0.56 ± 0.06, *p* = 0.002).

To confirm that the more complex model was not overfitting the data, we evaluated cross-validated data likelihoods (Methods). Again, the cross-validated likelihoods were highest for the model that incorporated both biases as compared to either the perceptual bias or baseline models (cvL_baseline_ = −562.6 ± 8.8, cvL_perceptualBias_ = −561.4 ± 18.9, cvL_bothBiases_ = −513.3 ± 13.3) (Fig 5B, right). The both biases model successfully fit all 10/10 participants’ data (median gof *p*-value: 0.87): the predicted responses closely matched the observed responses in the model that incorporated both biases (r_bothBiases_ = 0.96, *p* < 0.001) (Fig 5D) as compared to the other 2 models (r_baseline_ = 0.45, r_perceptualBias_ = 0.44, S5A and S5B Fig).

A model that incorporated only choice bias—and no perceptual bias—was only marginally worse than the both biases model (AICc_choiceBias_ = 1,034.1 ± 26.3; cvL_choiceBias_ = −514.4 ± 13.3; median gof *p*-value: 0.83). Yet, as compared to the “both biases” model, median AICc for the “choice bias” model was higher by 3.45 points, cross-validated likelihoods were worse for 7/10 participants, and goodness-of-fit *p*-values were poorer for 7/10 participants, suggesting that modeling perceptual bias (along with choice bias) contributed to improving model fit to behavior.

In sum, Bayesian analysis with the VP model revealed the role of perceptual and choice biases in mediating the behavioral consequences of presaccadic selection. Although the perceptual recency bias was useful for explaining presaccadic selection’s effects on sensitivity, choice bias was arguably the single most important factor for presaccadic effects on criteria, and significantly improved model fits to behavior. Taken together, our results pinpoint dissociable mechanisms by which perceptual and decisional processes mediate presaccadic selection. Moreover, they provide a parsimonious account for why presaccadic benefits—reported widely in visual discrimination tasks—are surprisingly absent during visual change detection.

## Discussion

Planning to move the eyes changes the way we perceive our visual world [2,3,12,25,45,47–50]. Many previous studies have employed elegant task designs to show that presaccadic attention enhances visual discrimination accuracy immediately before the eyes move; several more studies have proposed specific behavioral and neural mechanisms underlying these benefits [6,15–17,48–50]. Yet, no study, to our knowledge, has systematically explored the effects of presaccadic selection on spatial choice bias.

In many previous studies of presaccadic attention [7,9,27], a single discrimination target was displayed among distractors without an explicit location probe; such a design precludes inferring the location of the specific stimulus that biased the participant’s report. Alternatively, analysis of visual discrimination tasks with conventional signal detection theory models can only quantify biases for different stimulus features (e.g., clockwise versus counter-clockwise orientations). By contrast, the change detection and localization task design employed in our study—along with analysis with a multidimensional signal detection theory model [34]—enabled precisely decoupling and quantifying location-specific effects of presaccadic selection on perceptual sensitivity and spatial choice bias: While, choices were biased toward the saccade target location spatially, no improvement in change detection sensitivity occurred at this location presaccadically.

We explored this surprising result further with a second task that involved estimating the orientation of one of 2 Gabor patches, presented in quick succession, presaccadically. While presaccadic attention did enhance the precision of orientation estimation, this benefit was observed only for the most recent stimulus at the saccade target location. Moreover, the results revealed evidence for a novel “presaccadic recency bias”—a bias in orientation reports of the initial stimulus, induced by the final stimulus, at the saccade target location.

The presaccadic recency bias could readily explain the absence of change detection sensitivity benefits at the saccade target location. Because the perceived orientation of the initial stimulus was biased towards the final stimulus, accurate computation of the orientation change would be compromised at this location. Yet, the enhanced precision for the final stimulus possibly compensated for this detriment in the change signal computation. The net effect was, therefore, a lack of enhancement in change detection sensitivity at the saccade target location, relative to the other locations.

At first glance, the recency bias appears to be at odds with our finding regarding criteria at the saccade target location: Because of the recency bias, the perceived difference in orientation between the initial and final Gabors will always be smaller in magnitude than the actual difference. This should yield fewer false alarms, and a higher criterion, whereas we observed the converse—a lower criterion—at the saccade target location. These observations could be reconciled if we posit that in addition to the perceptual recency bias, presaccadic selection also produced a decisional (choice) bias—that reduced the decision criterion—at the saccade target location. This choice bias overwhelmed the effects of the perceptual bias, thereby yielding the highest proportion of false alarms at the saccade target location.

We tested this hypothesis using a Bayesian variable precision model. The variable precision model simulates trial-wise variations in internal estimates of orientation, based on which a Bayesian ideal observer would perform change detection and localization. In our case, the model also offered an elegant way of modeling both change detection and estimation tasks within a single framework. The VP model variant that best fit behavior was one that not only accounted for differences in precision across locations but also incorporated both a choice bias (lower detection threshold) and an attractive presaccadic recency bias at the saccade target location. In addition, given the potential for overfitting with the multi-parameter VP model, we estimated cross-validated data likelihoods using behavioral predictions of left-out sessions, which confirmed the robustness of these findings.

An alternative explanation for the recency bias we observed is an illusory “temporal order reversal” effect that occurs for sequences presented presaccadically [45,46]. We tested, and discounted, this explanation. For one, this reversal effect has been described only for highly transient stimulus sequences (approximately 10 ms flashes separated by 100 ms), and for reflexive saccades. Given that, in our task, the first stimulus set was displayed on screen for >1,000 ms, and the participants made volitional saccades, such a temporal order reversal is unlikely to have occurred. Second, the effect of temporal reversal has been described perisaccadically, in a time window spanning ± 50 ms locked to saccade onset. In our experiments, even after excluding trials within this range, we continued to observe the presaccadic recency bias (S4A Fig). Finally, while reports for the initial Gabor stimulus orientation were indeed closer to the final stimulus orientation than their own, the converse was not true; these results demonstrate clearly that participants were not mistakenly interchanging the identities of the initial and final Gabors.

Similarly, the lower criterion at the saccade target location could not be readily explained by spatial mislocalization biases arising from a perisaccadic compression of visual space [20,49]. Presaccadic mislocalization magnitudes up to approximately 10 dva have been reported along the saccade direction [20] and up to approximately 3 dva in the saccade orthogonal direction [22]. Yet, we observed no significant bias in change mislocalization rates regardless of where the change occurred relative to the saccade target. This finding is attributable to a few factors. First, in our task, the Gabor stimuli were fairly large (2.35° in radius) and were separated by 8.2° of visual angle from each other both along the azimuthal and the elevational directions. Moreover, in our task, saccade amplitudes were only approximately 4°, on average. We suspect that the magnitude of spatial mislocalization across our large and well-separated stimuli was relatively modest and, therefore, did not contribute significantly to mislocalization biases towards the saccade target.

Criterion modulations in our change detection tasks peaked in windows closest to saccade onset, providing clear evidence for a presaccadic effect. Moreover, we did not observe strong evidence for saccade resetting due to the transient associated with the blank and change that occurred during the presaccadic epoch. These results appear at odds with previous literature, which suggests that presaccadic sensory transients can induce saccade program resetting [40–43]. In these studies, highly salient—large and high-contrast, flashed—distractors were employed to demonstrate clear evidence for saccadic inhibition or resetting; such resetting effects typically occurred in a window beginning 50 to 70 ms following the flash [42]. We speculate that comparatively subtle sensory transient could explain the relative lack of saccadic inhibition effects in our task: Gabor stimuli were presented at only 30% peak contrast on a gray background and disappeared to background contrast for 20 ms before reappearing and disappearing again. This produced a localized “flicker”-like percept rather than a salient “flash”-like percept, which perhaps diminished the strength of saccade resetting. Nevertheless, to address the possibility that saccades were inhibited albeit to a lesser extent, we reanalyzed data by including only a subset of trials that likely escaped saccade resetting; robust criterion modulations occurred in these trials also. Together, these results indicate that criterion changes observed in our study are a consequence of presaccadic selection mechanisms.

The presaccadic selection effects reported in our study likely reflect a mixture of prioritization in encoding and iconic memory at the saccade target location. In the orientation estimation and change detection tasks, the saccade cue appeared even while the initial stimulus set was present on the screen. Moreover, for the vast majority of trials (>95%), the saccade itself was executed <230 ms after either set of stimuli had disappeared; this presaccadic epoch is well within the range of iconic memory [51,52]. Another recent study showed that saccades induce prioritization in visual short-term memory [53], with saccade cues that were presented 100 to 3,200 ms after stimulus offset. It is possible that the mechanisms underlying presaccadic benefits reported in shortest intervals (approximately 100 ms) of this previous study [53] are similar to those reported in our study. Yet, importantly, our study quantified the precision of orientation estimates for multiple, sequentially presented stimuli in the presaccadic epoch in “double set” trials, with the goal of explaining the results of the change detection task. Our results revealed a systematic bias in the estimates of first (initial) stimulus orientation toward the second (final) stimulus.

Nevertheless, because orientation estimates were reported at the end of each trial in our task, presaccadic prioritization that occurred during encoding could have continued into visual short-term memory. Is it possible, then, that the presaccadic recency bias was a result of interference between visual-short term memory representations? We consider this explanation unlikely. In double set trials, observers were required to encode and maintain only one set of stimuli—either the initial or the final set—in a blocked design. In 50% of the blocks, they were explicitly informed, a priori, that the final stimulus set was task irrelevant. Yet, even in these blocks, we observed a robust recency bias occurred at the saccade target location (Fig 3B and 3C). In addition, this bias did not occur at any of the other 3 (saccade away) locations. Thus, this space-specific bias at the saccade target location is a direct consequence of presaccadic selection mechanisms.

Our results add to a growing body of literature that investigates shared and distinct mechanisms mediating covert endogenous attention and presaccadic attention. A few previous studies [6,54] employed a saccade cue that was 100% predictive of the location of the upcoming discriminandum; such a design may conflate the effects of presaccadic attention with those of covert endogenous attention. But in our study, the saccade cue was entirely uninformative about the upcoming location of change; this design enabled us to distinguish the effects of presaccadic attention from those of endogenous attention. Whereas endogenous attention has been reported to enhance both change detection bias and sensitivity at the location cued for attention [35,37,55,56], we show that presaccadic selection enhances change detection bias, but not sensitivity, at the saccade target location. Given that we employed a task design with stimulus configuration and timings closely similar to previous endogenous attention studies, we propose that these differences in behavioral effects between these types of attention arise due to the unique nature of the presaccadic recency bias. While the recency bias occurred in this presaccadic selection task—and impacted change detection sensitivity—no such bias has been reported in endogenous attention tasks.

Along similar lines, our results appear at odds with previous studies on trans-saccadic change blindness [57,58]. Such studies have shown that trans-saccadic changes in key object features (e.g., position, luminance, or size) that occur in the direction of the saccade are detected better than changes that occur in the direction opposite [57–59]. Yet, these studies do not distinguish the effects of presaccadic attention from those of endogenous (voluntary) attention; the latter is typically directed toward the location of the impending saccade [2,3,60] and could facilitate the detection of such trans-saccadic changes. On the other hand, we controlled for the confounding effects of endogenous attention in our task by employing an uninformative saccade cue and a brief (<300 ms) presaccadic window. Our results demonstrate that presaccadic selection mechanisms, by themselves, do not yield sensitivity benefits in change detection.

Our results motivate the search for the neural bases of the distinct presaccadic effects on sensitivity and bias. Recent studies suggest that a presaccadic shift and scaling of visual receptive fields at the saccade target may underlie the enhanced visual discrimination sensitivity prior to saccades [47,49,61,62]. Furthermore, visual area V4 neurons have been shown to preferentially encode the stimulus features at the saccade target [48]. Such a mechanism may mediate the advantage in precision we observed for the most recent stimulus at the saccade target location. Moreover, several studies have reported a presaccadic gain modulation in neural responses in the visual area V4 [47,50] and frontal eye fields [49]. Such a gain-modulation is a putative neural mechanism mediating the increase in presaccadic choice bias at the saccade target location. Notably, similar bias (criterion)-related neural gain modulations have been reported in visual cortex, recently [63].

The timing of our presaccadic effects is consistent with previously reported neurophysiological correlates of presaccadic selection. For example, Moore and Chang (2009) [48] showed that firing rate modulations and neuronal discriminability in area V4 commenced around 150 ms prior to saccade onset and peaked around 50 ms prior to the saccade. Some criterion effects in our data may reflect space-specific differences in other saccade-related neural processes, such as saccadic suppression. While some studies have suggested that saccadic suppression effects differ across locations [64,65], other studies have reported no such differences [66,67]. We observed strong and significant criterion differences in 2 windows (0–50 ms and 50–100 ms) immediately preceding the saccade. It is likely that the earliest of these windows (0–50 ms) is subject to saccadic suppression (gray shading, Figs 1G–1J and S1B). Yet, accounting for visual cortical response latencies (approximately 50 ms) [68–69]—the time between stimulus presentation and this information reaching early visual cortex—we propose that presaccadic selection effects are best characterized in the penultimate presaccadic (50–100 ms) window. Interestingly, neither of these windows showed presaccadic sensitivity effects, suggesting that saccadic suppression-linked mechanisms alone are unlikely to explain these findings.

Our study can be readily extended in several directions. While we report an all-or-none presaccadic benefit that occurs entirely for the last of 2 stimuli, and not at all for the first, it would be interesting to know if the effect occurs in an all-or-none or graded manner when a sequence of multiple stimuli is presented presaccadically. Furthermore, investigating presaccadic recency bias in a naturalistic setting (e.g., with virtual reality displays) may inform our understanding of how a stable percept of the world emerges despite frequent eye movements in free-viewing, real-life conditions.

## Materials and methods

### Participants

A total of 21 unique participants (9 females, age range: 19 to 36 years, median age: 24 years, all right-handed), with normal or corrected-to-normal vision, and no known history of neurological disorders participated in the experiments; one of the authors (PG) also participated. Ten participants (6 females, age range: 19 to 27 years) performed the orientation change detection task. Seven participants (2 female, age range: 19 to 27 years) performed the contrast increment detection task. Ten participants (4 females, age range: 20 to 36 years) performed an orientation estimation task. Four participants performed both the orientation change detection and contrast increment detection tasks. One participant (2 participants) performed both the orientation change detection (contrast increment detection) and orientation estimation tasks. Experimental protocols were approved by the Institute Human Ethics Committee (IHEC), Indian Institute of Science (IISc), Bangalore (approval no.: 3–21082020). Informed written consent was obtained from all participants.

### Experimental design

#### Behavioral tasks

*Setup*. Participants performed the tasks in a dimly illuminated room, with their head stabilized on a chin and forehead rest, seated 60 cm from a contrast-calibrated stimulus display (BenQ XL2411Z, 100 Hz refresh rate). The task was designed with custom scripts using Psychtoolbox (version 3.0.15) implemented on MATLAB 2015b. Gaze position was tracked with an infrared eyetracker (SMI iViewX HiSpeed, SensoMotoric Instruments) at a 500 Hz sampling rate; analyses of gaze position were performed both online and offline (see next).

*Orientation change detection and localization task*. Participants performed a dual task, comprising detecting and localizing orientation changes at one of 4 peripheral Gabor patch stimuli (4-ADC task [34]), while also planning and executing saccades to one of the 4 stimulus locations. A trial began with fixation on a central dot (positive contrast, 0.3° diameter) concurrently with the presentation of 4 circular placeholders (positive contrast circles, 5.3° diameter), one in each visual quadrant at an eccentricity of 5.8° along the diagonal. Centered within each placeholder a Gabor patch (30% peak contrast, Gaussian SD: 0.8°, spatial frequency: approximately 1.9 cycles/°, aperture diameter: 4.7°) was presented. Gabor orientations were drawn from circular uniform distributions, independently of each other. Following 1,000 ms of this initial stimulus set, a saccade cue (line segment, negative contrast, radial extent: 0.3°) adjoining the central dot appeared for 50 ms, pointing towards one of the quadrants. Participants were instructed to make a saccade towards the center of the stimulus in the cued quadrant (“saccade target”) as accurately and as soon as possible following saccade cue onset. Following saccade cue onset, the initial stimulus set remained on the screen for a variable, additional interval drawn from a geometric distribution (10 to 210 ms, in steps of 20 ms). Following this, the stimuli disappeared for 20 ms, and reappeared for 20 ms with either one or none of the Gabor stimuli having changed in orientation. Upon successful completion of the saccade (gaze position crossing the appropriate placeholder boundary), a set of 5 response boxes appeared on the screen in a linear row (Fig 1A). Participants indicated the location at which they perceived a change in orientation or “no change” by clicking the appropriate response box with an optical mouse (4,000 ms response window). This linear layout of response options was chosen so as to avoid systematic spatial correspondences between the location of the response buttons and the saccade target location, either in terms of left versus right, or upper versus lower, hemifield locations (Fig 1A); the goal was to avoid response biases due to eye-hand coordination—which may occur if the location selected for oculomotor response were aligned with the subsequent manual response. The response boxes also appeared if no successful saccade was recorded within 400 ms of saccade cue onset; such trials (mean ± SD across participants: 11.9% ± 8.9%) were discarded before subsequent analyses. The saccade cue was not informative about the location of change: the change was equally likely to occur at any of the 4 locations (20% trials), or not at all (20% trials). Participants were informed about these task design elements, a priori.

The experiment was conducted over 2 days. On the first day, participants were trained on the 4-ADC task with a 90° change angle, without a saccade cue, but with feedback on their accuracy on each trial. Following this, the change angle was staircased for each participant to approximately 55% accuracy, also with the no-saccade 4-ADC task (mean ± SD: 17.70 ± 4.08°). Following the staircasing, participants were trained on the dual task paradigm in which they performed the change detection along with saccade execution and were provided with feedback for both task objectives. This training was typically performed for 75 to 150 trials, with the staircased change angle.

On day 2, in a preliminary training session, participants performed 1 to 4 blocks of 75 trials each, after which testing commenced. The testing phase comprised 8 blocks of 75 trials without feedback as part of the main experiment. In a subset of 5 participants, we measured the psychometric function with 4 change angles (10°, 25°, 40°, and 55°); these sessions, comprising 800 trials per participant, were acquired on a separate experimental day. Data from the training blocks were not included in the subsequent analyses.

*Contrast increment detection and localization task*. Participants were tested on a dual task, as before, except that they detected and localized a contrast increment at one of 4 Gabor stimuli while also planning and executing saccades to one of the 4 stimulus locations. The task design was similar to the orientation change detection task, except for a few differences in stimulus configuration. First, all of the Gabor stimuli were vertically oriented, and their contrasts were drawn, randomly and uniformly, from 4 non-overlapping contrast bins ([10% to 20%], [30% to 40%], [50% to 60%], [70% to 80%]). Second, when the stimuli reappeared after the first blank (Fig 1A, bottom), the contrast of one or none of the stimuli was incremented by 20%. Participants were instructed to indicate the location of perceived increase in contrast, or no change, as before, by clicking on one of 5 response boxes. Finally, staircasing was performed by varying the duration of the final set to achieve approximately 55% accuracy (30 to 60 ms across participants).

*Orientation estimation task*. Participants performed a dual task, as before, except that they estimated the orientation of one of 4 Gabor stimuli while also planning and executing saccades to one of the 4 stimulus locations. Stimulus sequence and timings were nearly identical with the orientation change localization task except that the initial and final Gabor orientations at each location were drawn independently from a circular uniform distribution (Fig 2A, middle). In addition, the initial stimulus set duration was drawn from a geometric distribution (10 to 210 ms, steps of 100 ms). In distinct blocks of trials, participants estimated the orientation of the probed Gabor either from the initial or from the final set of stimuli; written instructions regarding the set being probed (“initial set probed” or “final set probed”) were provided onscreen at the beginning of each block. At the end of the trial, a central response probe (positive contrast central dot, 0.5° diameter, with negative contrast quadrant) indicated the location of the Gabor stimulus for response. Upon moving the mouse cursor, a randomly oriented gray bar (2.9° length, 0.3° thickness) appeared behind the central probe. Participants were instructed to rotate the response bar until the bar’s orientation matched that of the probed Gabor and clicked the left mouse button to complete their response. Participants were afforded 1,500 ms to initiate and 2,500 ms to complete their response. As with the previous tasks, the saccade cue was uninformative regarding the location of the probed Gabor grating.

In 60% of trials, Gabor stimuli were presented both as the initial and final set of stimuli “double set” trials. In 1 group of (*n* = 5) participants, in 40% of trials interleaved with the double set trials, only the initial set of Gabor stimuli were presented, and the second set of Gabor stimuli were replaced with a set of blanks (“single set” trials). In the remaining (*n* = 5) participants, in 40% of trials, interleaved with the double set trials, the second set of Gabor stimuli were replaced with filtered noise masks (“noise mask” trials). Noise masks were generated by bandpass filtering Gaussian noise (mean background contrast, SD: 16.7% contrast) in a band from 0.5× to 2× the spatial frequency of the Gabor stimuli [6]. Because only 1 set of Gabor stimuli were presented in both single set and noise mask trials, for these trials participants were instructed to report the orientation of whatever Gabor stimulus they perceived at the probed location, regardless of block type (initial set probed or final set probed).

*Eyetracking and trial exclusion*. Gaze position was calibrated with a 9-point calibration (iViewX) at the beginning of the experiment, and periodically throughout the experiment. Saccade onsets were detected offline with the BeGaze software based on the following criteria: gaze deviation from fixation >2°, velocity >50°/s, duration >8 ms, and confirmed with manual inspection. For subsequent analyses, the following stringent criteria were used for trial inclusion based on gaze dynamics: (i) the saccade must be made to the correct quadrant indicated by the saccade cue; (ii) gaze position must cross the placeholder boundary within 400 ms of saccade cue onset; and (iii) stable fixation (<1° change in gaze position) from saccade cue onset, until saccade onset. In addition, we excluded those trials in which the saccade onset occurred before the second set of stimuli had disappeared. Based upon these criteria, the trial inclusion rates for the different tasks were as follows (mean ± SD, across participants): (i) orientation change detection and localization: 78.7% ± 6.4%; (ii) contrast increment detection and localization: 73.7% ± 6.7%; and (iii) orientation estimation: 64.8% ± 7.2%.

### Data analyses

#### Change detection and localization tasks

For the change detection and localization tasks, we estimated psychometric and psychophysical parameters at the ST and SA locations. The same analysis approach was used for both the orientation change detection and the contrast increment detection tasks.

*Constructing contingency tables*. Prior to signal detection theory analysis, we constructed 5 × 5 stimulus-response contingency tables for each participant using all trials from each experimental session (Fig 1D, left). Rows of this table represent the 5 potential stimulus events (change at each of the 4 stimulus locations, or no change), whereas columns represent the 5 possible responses (report of change detected at each of the 4 locations, or no change). For these analyses, trials were sorted so that the first 4 rows of the table represented the location of the change relative to the saccade cue: cued (location towards which the saccade cue pointed), opposite (location diametrically opposite to the cued location), ipsilateral (location in the same hemifield as the cued location), and contralateral (location in the opposite hemifield as the cued location). We refer to the cued location as the “Saccade Toward” or “Saccade Target” (ST) location, and the opposite, ipsilateral and contralateral locations collectively as the “Saccade Away” (SA) locations. Similarly, the first 4 columns were re-sorted so that they represented the participant’s response location relative to the saccade cue. Then, contingency table encodes response proportions for multiple different response types (Fig 1D, left). The values along the diagonal encode correct responses: hits (H), when the participant reported the location of change accurately and correct rejections (CR) when the participant correctly reported no-change. The off-diagonal elements encode 3 kinds of incorrect responses: misses (M) when the participant reported no-change even when a change occurred (last column), false alarms (FA) when the participant reported a change on no-change trials (last row), and mislocalizations (ML), when participant incorrectly localized the change (all other off-diagonal elements).

*Estimating psychometric and psychophysical parameters*. Psychometric quantities (hit rates and false alarm rates) for each location were computed from the contingency tables. For orientation change detection with multiple change angles (*n* = 5 participants), we also computed the psychometric function by fitting a three-parameter sigmoid curve to the hit rate as a function of change angle.

To estimate the psychophysical parameters (sensitivity and criteria), we employed a multidimensional signal detection model (the m-ADC model; Fig 1D right), developed specifically for the analysis of behavioral data in multialternative detection tasks [34]. The model has been tested and validated widely for analyzing many kinds of detection tasks, both with humans and with nonhuman primates [33,35–37,70]. A detailed description of the model is available in these previous studies; we present a brief description here. In the 4-ADC model for our task the decision is modeled as multivariate Gaussian random variables. The decision variable distributions representing sensory evidence for change (signal) at each the 4 locations are represented along orthogonal axes. The decision variable distribution corresponding to no-change (noise) is centered at the origin. The mean of the signal distribution along each axis varies with the sensitivity (d’) for detecting change at that location. The decision boundary is parameterized by 4 decision thresholds (t), one for each location. On each trial, the observer indicates a change at the location at which the decision variable exceeded the corresponding threshold by the largest value. If the decision variable did not exceed threshold at any location, the observer indicates a “no-change.” We derive the criterion measure of choice bias as (c = t– d’/2) [33,71]. The criterion is inversely related to choice bias so that the lower the criterion at a location, the higher the choice bias towards that location.

Each psychophysical parameter is mathematically related to observed response proportions—hits, correct rejections, misses, false alarms, and mislocalizations (Fig 1D)—by modeling observers’ choices in a multidimensional decision space (see [33,34] for the full mathematical relationship). Although the association between d’ (or c) and observed response proportions is arguably more complex in a multialternative change detection task than in a simple change detection task, some relationships continue to hold across task types. For example, the lower the criterion at a location relative to other locations, higher is the probability of reporting a change at that location. In other words, a lower criterion at a location yields a higher proportion of at least one of the following responses—hits, mislocalizations, or false alarms—at that location. Or, the higher the sensitivity at a location, the higher the proportion of at least one of the following responses—hits or correct rejections—at that location. The m-ADC model was fit to response proportions in the stimulus-response contingency table, and sensitivity and criteria were computed using maximum likelihood estimation. Goodness-of-fit, estimated with a randomization test based on the chi-squared statistic, indicated that model fits did not deviate significantly from the data (*p* = 0.6 [0.1–1.0], median [range]).

In the results in the main text, we compared psychophysical parameters at the ST location with the average values across the 3 SA locations; we also performed pairwise comparisons between the saccade-target ipsilateral and contralateral locations with a Bonferroni–Holm correction for multiple comparisons. To confirm that the observed modulation in criterion was not a direct consequence of peri-sacccadic mislocalization, we re-estimated psychophysical parameters using a one-dimensional signal detection theory model [72] after excluding all mislocalizations from the stimulus-response contingency tables.

We also estimated the temporal dynamics of presaccadic selection’s effects on psychophysical parameters. For this analysis, we binned trials with similar corrected intervals between final set onset and saccade onset, in non-overlapping bins of 50 ms duration, centered at –175, –125, –75, and –25 ms before saccade onset. Psychophysical parameters were estimated by constructing contingency tables for each time window. For analyzing the temporal dynamics of the sum of hit and false alarm rates, we binned trials finely (width: 50 ms, shift: 5 ms), averaged these values across participants, and plotted them aligned to saccade onset (200 ms before to 50 ms after saccade onset; S1B Fig).

#### Orientation estimation task

For the orientation estimation task, we computed the orientation report error at the ST and SA locations. Performance was quantified with the mean absolute error (MAE): the absolute deviation (in degrees) of the reported orientation from the true orientation of the probed stimulus, averaged across trials. For the double set trials, we analyzed each block type—initial set probed, and final set probed—separately. For the single set and noise mask trials, we report results from only the initial set probed blocks, because the Gabor stimuli for these trials always occurred in the initial set. Essentially, for these trials we sought to avoid detrimental effects (e.g., due to mismatched temporal expectation) on orientation estimates of a Gabor stimulus presented in the initial set in a block in which the final set was consistently probed. Nonetheless, pooling behavioral responses from both block types yielded nearly identical results. We computed precision as the reciprocal of the non-parametric circular standard deviation [73] of the mean-subtracted, signed estimation error distributions. In Fig 2B–2E, for visualization, we also plotted the von Mises function with concentration parameter fit using maximum likelihood estimation (data pooled across participants).

To quantify the influence of the final set on the report of initial set (recency bias) and vice-versa (primacy bias), we computed “bias” for the probed stimulus relative to the biasing stimulus using the signed area under the curve [74,75]. Briefly, we binned trials according to the orientation of the biasing stimulus relative to the orientation of the probed stimulus (bin width = 60°, sliding by 1°) and plotted the median estimation error in these trials for each bin, to obtain a response bias curve. We computed the signed area under this curve, in the range of 10° to 60° magnitude (positive and negative) of relative orientation of the biasing orientation to the probed orientation. A positive signed area indicates an attractive bias toward the biasing stimulus (higher area in the first and the third quadrants), whereas a negative signed area indicates a repulsive bias away from the biasing stimulus (higher area in the second and the fourth quadrants).

#### Fitting behavior with the variable precision model

We modified the VP model to fit participants’ behavior in the multialternative change detection task. A detailed description of the standard VP model is available in van den Berg and colleagues [38]; in our results, we call this the “baseline” model (Fig 5A). Briefly, the VP model assumes that precision varies across items and trials. On each trial, the value of precision (*J*) at each location is drawn from a gamma distribution, parameterized by mean ($\bar{J}$) and scale (*τ*) parameters for that location, i.e., $p(J|\bar{J};\tau )=\mathrm{Gamma}(J;\bar{J},\tau )$. The internal measurement *x* of an external stimulus *θ* on each trial is assumed to follow a von Mises (VM) distribution, centered on *θ* and with a concentration parameter *κ* which determines the variance of the noise in measurement; the variance is inversely proportional to *κ*. The relationship between the concentration parameter, *κ* and precision, *J* is is denoted by *κ* = *ϕ*(*J*), and computed numerically. Therefore, the distribution of internal measurements is given by a mixture of VM distributions, with parameters drawn from the corresponding gamma distributions. Thus, the internal measurement is distributed as $p(x|\theta ;\bar{J},\tau )=\int p(x|\theta ;J)p(J|\bar{J},\tau )dJ=\int \mathrm{VM}(x;\theta ,\phi (J))\mathrm{Gamma}(J;\bar{J},\tau )dJ$. We approximated this distribution by averaging VM distributions corresponding to 1,000 samples of *J* (drawn from the gamma distribution). To incorporate motor noise during the report $p(x|\theta ;\bar{J},\tau )$ was convolved with another VM distribution with concentration parameter *κ*_*m*_; the latter parameter was fixed (*κ*_*m*_ = 25) based on its value in a previous study [38].

*Orientation estimation task*. Based on evidence from our experiments, we modeled different mean precisions $\bar{J}$ for the initial and final stimuli at the ST location (${\bar{J}}_{ST}$ and ${\bar{J}\prime }_{ST}$, respectively), and a common mean precision at the SA locations (${\bar{J}}_{SA}$). Along with these 3 parameters, we estimated a common *τ* (scale) across all locations. To ensure robust parameter estimates, we pooled the error distribution in the orientation estimation task across participants; this was done separately for the initial and final stimuli at the ST location but combined across stimuli at the SA locations. Model parameters were estimated with maximum likelihood estimation using grid-search by simultaneously fitting these 3 error distributions [38].

*Orientation change detection and localization task*. Internal measurements corresponding to the orientations of the initial (***θ***_***i***_) and the final (***θ***_***f***_) stimuli are represented by latent vectors (***x*** and ***y***, respectively); these follow von Mises distributions with concentration parameters ***κ***_***x***_ and ***κ***_***y***_, respectively. For example, for each location *l* in the initial stimulus set, the internal measurement *x*_*l*_ for each trial is drawn from a VM distribution with concentration parameter *κ*_*x*,*l*_, and centered at *θ*_*i*,*l*_. Similarly, for the final stimulus set, the internal measurement *y*_*l*_ is drawn from a VM distribution with concentration *κ*_*y*,*l*_ centered at *θ*_*f*,*l*_. Across trials, the concentration parameters vary depending on the values of precision *J* drawn from their respective gamma distributions. At the change location *c*, *θ*_*i*,*l* = *c*_ and *θ*_*f*,*l* = *c*_ differ by the change angle Δ*θ*. At the other locations, change angle was zero as per our actual experimental task design, so that *θ*_*i*,*l*≠*c*_ = *θ*_*f*,*l*≠*c*_. The posterior odds ratio of change to no-change at each location $\frac{p(l=C|x,y)}{p(l=N|x,y)}$ evaluates to a quantity proportional to $F(l_{C})=\frac{I_{0}(\kappa_{y})I_{0}(\kappa_{y})}{I_{0}(\sqrt{\left(\kappa_{x}^{2}+\kappa_{y}^{2}+2\kappa_{x}\kappa_{y}\cos\left(y-x\right)\right)})}$, where *I*_0_ is the modified Bessel function of the first kind of order zero [76]. To model change localization along with a no change response, we introduced a “decision threshold” parameter *η* in the model. If the posterior odds ratio does not exceed the decision threshold for any location *l*, the observer reports a “no change.” Otherwise, the observer reports a change at the location at which maximally exceeds the decision threshold at that location (argmax [*F*(*l*_*C*_)−*η*]).

We chose constraints on the model informed by the empirical results observed in the orientation estimation task. First, we modeled a common mean precision parameter for the initial stimulus at the ST location and the stimuli at the SA locations, because the mean precision estimates were comparable in the orientation estimation task paradigm. Second, because the mean precision for the final stimulus at the ST location was estimated to be 6 times the mean precision for the initial stimulus, we scaled the mean precision for the final stimulus at the ST location accordingly. Third, we used a common scale parameter, *τ*, across locations and stimuli, with value estimated from the orientation estimation task.

In the “baseline” model, the decision threshold, *η*, was fixed to 1. In the “perceptual bias” model, in addition, we modified the model to include a presaccadic recency bias *B* at the ST location. The internal measurement for the initial ST stimulus was modeled with a two-step process. First, an intermediate measurement *x*_*ST*_ was computed with VM noise with *κ*_*x*,*ST*_. Next, to obtain the final measurement *x*′_*ST*_, we modeled a perceptual bias towards final ST stimulus (*y*_*i*_) as: ${x\prime }_{ST}={By}_{ST}+(1-B)x_{ST},B\in [0,1]$. In the “both biases” model, we additionally modeled differential thresholds at the ST and SA locations, which were estimated during model fitting. Finally, the “choice bias” model incorporated these differential thresholds as in the “both biases” model, but not a perceptual bias. Thus, the numbers of parameters for the baseline, perceptual bias, choice bias and both biases models were 1, 2, 3, and 4, respectively. The distribution of the maximum a posteriori (MAP) estimate, $p(\hat{C}|C,z,\kappa_{x},\kappa_{y})$, was computed through Monte Carlo simulation with 1,000 samples of *J* and 4,800 samples of ***x*** and ***y***.

Model fits were performed for each participant individually, with responses averaged across the SA locations. As before, model parameters were estimated with maximum likelihood estimation (multinomial likelihoods) using generalized pattern search. For formal model comparison, we employed the corrected AICc [77], estimated independently for each participant. Goodness-of-fit was evaluated with a randomization test based on the chi-squared statistic. For 2/10 participants for whom the goodness-of-fit *p*-value suggested poor model fits (<0.05) for the both biases model, we re-ran the parameter estimation with an additional initial condition; this same initial condition was then tested across the choice bias model also. To avoid the potential for overfitting with the more complex models, we computed cross-validated data likelihoods using a 4-fold cross-validation using a “leave session out” approach. Each model’s predicted response proportions, obtained from the left out fold, were compared against observed response proportions using Pearson’s correlations.

### Statistical analyses

For change detection and localization tasks (both orientation and contrast), we employed a permutation test to test for significance of differences in sensitivity and criterion across the ST and SA locations (delta d’ or delta c), by comparing the observed differences against null distributions of differences. The null distributions were obtained by random shuffling of location labels 1,000 times for comparisons (“random” permutation test).

For estimating significant differences in the analysis of temporal dynamics of sensitivity and criterion, we employed an identical permutation test, except that significance was evaluated independently in 4 non-overlapping 50 ms duration windows centered at –175, –125, –75, and –25 ms with respect to saccade onset. Here, and elsewhere, unless stated otherwise, multiple comparisons correction was performed with the Bonferroni–Holm procedure. For comparison across different SA locations, we used a Kruskal–Wallis test. For the analysis that involved comparing mislocalizations at the ST when change occurred at SA with mislocalizations at SA when change occurred at ST, we used a permutation test with shuffling of toward and away location labels. For computing significant differences in the temporal dynamics of the proportion of hits and false alarms, we used a cluster-based permutation test [78] across time and have reported the lower bound of the first bin of the significant cluster.

For orientation estimation, all the tests of significance for differences in estimation error, across the Saccade Toward and Saccade Away locations were also performed using a permutation test. For comparisons with *n* = 5 participants (single set and noise mask trials), and for comparisons with *n* = 10 participants (double set trials) either permutation or random permutation tests were employed; these are indicated in the Results, where appropriate. For bias, we tested for significant difference from zero using a two-tailed Wilcoxon signed rank. Similarly, for comparing estimated model parameters across locations and conditions, we employed a non-parametric two-tailed Wilcoxon signed rank test.

Finally, we also computed BF based on the Jeffreys-Zellner-Siow (JZS) prior [79] for pairwise *t* tests (change detection tasks: one-tailed for criterion and sensitivity; orientation estimation tasks: one-tailed for estimation error and two-tailed for bias) in all pairwise comparisons between behavioral metrics at the ST and SA locations [80]. BF > 3 (or >10) reflects substantial (or strong) evidence favoring the presence of the effect of interest. In addition, we examined how strength of evidence based on the BF changed as successive data points were collected, using Bayesian Sequential Analysis with the JASP software [81]. This analysis was performed for d’ and criterion for the visual change detection tasks, as well as precision for the initial and final stimuli, as well as the primacy and recency biases, for the orientation estimation task.

## Supporting information

S1 Fig Presaccadic selection effects in different time windows and for different stimulus strengths. **(A)** Relationship between estimated criterion (y-axis) plotted against proportion of hits and false alarms (x-axis), at the Saccade Toward (purple) and the Saccade Away (orange) locations. Distinct symbols denote distinct time bins prior to saccade onset. Circle: –300 to –200 ms, triangle: –200 to –100 ms, square: –100 to 0 ms. Each point denotes 1 time bin per participant. **(B)** (Top) Temporal dynamics of the sum of hit and false alarm rates at the Saccade Toward (purple) and Away (orange) locations locked to saccade onset (dashed vertical line). (Bottom) Presaccadic modulation of hit and false alarm rates locked to saccade onset (Δ = Toward–Away). Solid line: Mean values across participants, shaded error bars: SEM. Shaded gray zone: saccadic suppression epoch. Red horizontal line: cluster of significant differences. Other conventions are the same as in Fig 1E. **(C)** Criterion (top) and sensitivity (bottom) for orientation change detection reports at the Saccade Toward and Saccade Away locations, for trials with change onset <70 ms before the saccade onset. Other conventions are the same as in Fig 1E. **(D)** Same as in panel C, but showing criterion and sensitivity for trials with blank onset <70 ms before the saccade onset. **(E)** Same as in panel C, but showing criterion and sensitivity for trials with change offset <70 ms before the saccade onset. **(F)** Psychometric function of sensitivity at the Saccade Toward and Saccade Away locations for orientation change detection and localization with multiple change angles (10°, 25°, 40°, and 55°). Error bars: SEM (*n* = 5 participants). **(G)** Same as in Fig 1I, but showing temporal dynamics of difference in sensitivity at the Saccade Toward and Away locations for the contrast change detection task. **(H)** Same as in Fig 1J, but showing temporal dynamics of difference in criterion at the Saccade Toward and Away locations for the contrast change detection task. Data are available at https://dx.doi.org/10.6084/m9.figshare.21792002.
(TIF)

S2 Fig Transient-locked saccade onset times. **(A)** Same as in Fig 1C, but showing the distribution of saccade onset times locked to the time of transient (blank onset), pooled across participants, in the orientation change detection task. Annotation: *p*-values for Hartigan’s dip test of unimodality. Other conventions are the same as in Fig 1C. **(B)** Same as in panel A, but showing the distribution of saccade onset times locked to the time of transient for each participant (distinct plots). Data are available at https://dx.doi.org/10.6084/m9.figshare.21792002.
(TIF)

S3 Fig Bayesian Sequential Analysis robustness check. **(A)** Bayesian Sequential Analysis based on a paired sample *t* test, as a function of sample size, for d’ difference between the Saccade Toward (ST) and Saccade Away (SA) locations in the change detection tasks. Samples are pooled over the orientation change (*n* = 10) and contrast change (*n* = 7) detection experiments to yield a total sample size of *n* = 17. Here, H0 is the hypothesis that d’ is not different between the ST and SA locations while H1 is the alternative hypothesis that d’ is different across these locations. The x-axis represents the sequential sample sizes (from *n* = 1 to *n* = 17 participants), the left y-axis indicates the Bayesian Factor supporting H1 over H0 (BF_10_), and the right y-axis provides labels for different BF levels. Similarly, BF_01_ quantifies the evidence supporting H0 over H1. (Inset top-center) Pie chart indicates the data likelihoods under the 2 hypotheses (white: H0 and red: H1). **(B)** Same as in panel (A) but for criterion difference between the ST and SA locations in the change detection tasks. Other conventions are the same as in panel A. **(C)** Same as in panel (A) but for a difference in initial stimulus precision between the ST and SA locations, in “double set” trials, in the orientation estimation task (*n* = 10). **(D)** Same as in panel (C) but for a difference in final stimulus precision between the ST and SA locations, in “double set” trials, in the orientation estimation task (*n* = 10). **(E)** Same as in panel (A) but for recency bias at the ST location in the orientation estimation task (*n* = 10). **(F)** Same as in panel (E) but for primacy bias at the ST location in the orientation estimation task (*n* = 10). **(B–F)** Other conventions are the same as in panel A.
(TIF)

S4 Fig Control analyses for temporal order reversal effects. **(A)** Same as in Fig 3C (main text), but showing recency bias at the Saccade Toward and Saccade Away locations, following exclusion of trials in which the saccade onset occurred <50 ms after final stimulus offset. **(B)** Same as in panel A, but showing primacy bias at the Saccade Toward and Saccade Away locations. **(A, B)** Other conventions are the same as in Fig 3C. Data are available at https://dx.doi.org/10.6084/m9.figshare.21792002.
(TIF)

S5 Fig Model predictions of behavioral responses in the change detection task. **(A)** Same as in Fig 5E (main text), but showing response proportions predicted with the baseline model (y-axis) versus observed (true) response proportions (x-axis) in the change detection task. **(B)** Same as in Fig 5E (main text), but showing response proportions predicted with the perceptual bias model (y-axis) versus observed (true) response proportions (x-axis) in the change detection task. **(A, B)** Other conventions are the same as in Fig 5E. Data are available at https://dx.doi.org/10.6084/m9.figshare.21792002.
(TIF)

## Abbreviations

AICc: corrected Akaike information criterion
BF: Bayes factor
CR: correct rejection
FA: false alarm
m-ADC: multialternative detection/change-detection
MAE: mean absolute error
SA: Saccade Away
ST: Saccade Toward
VM: von Mises
VP: variable precision
