# Supplementary figures and images for: Presaccadic attention does not facilitate the detection of changes in the visual field
Source: PLoS Biol. 2024 Jan 25;22(1):e3002485. doi: 10.1371/journal.pbio.3002485 (PMC10810526; doi:10.1371/journal.pbio.3002485)

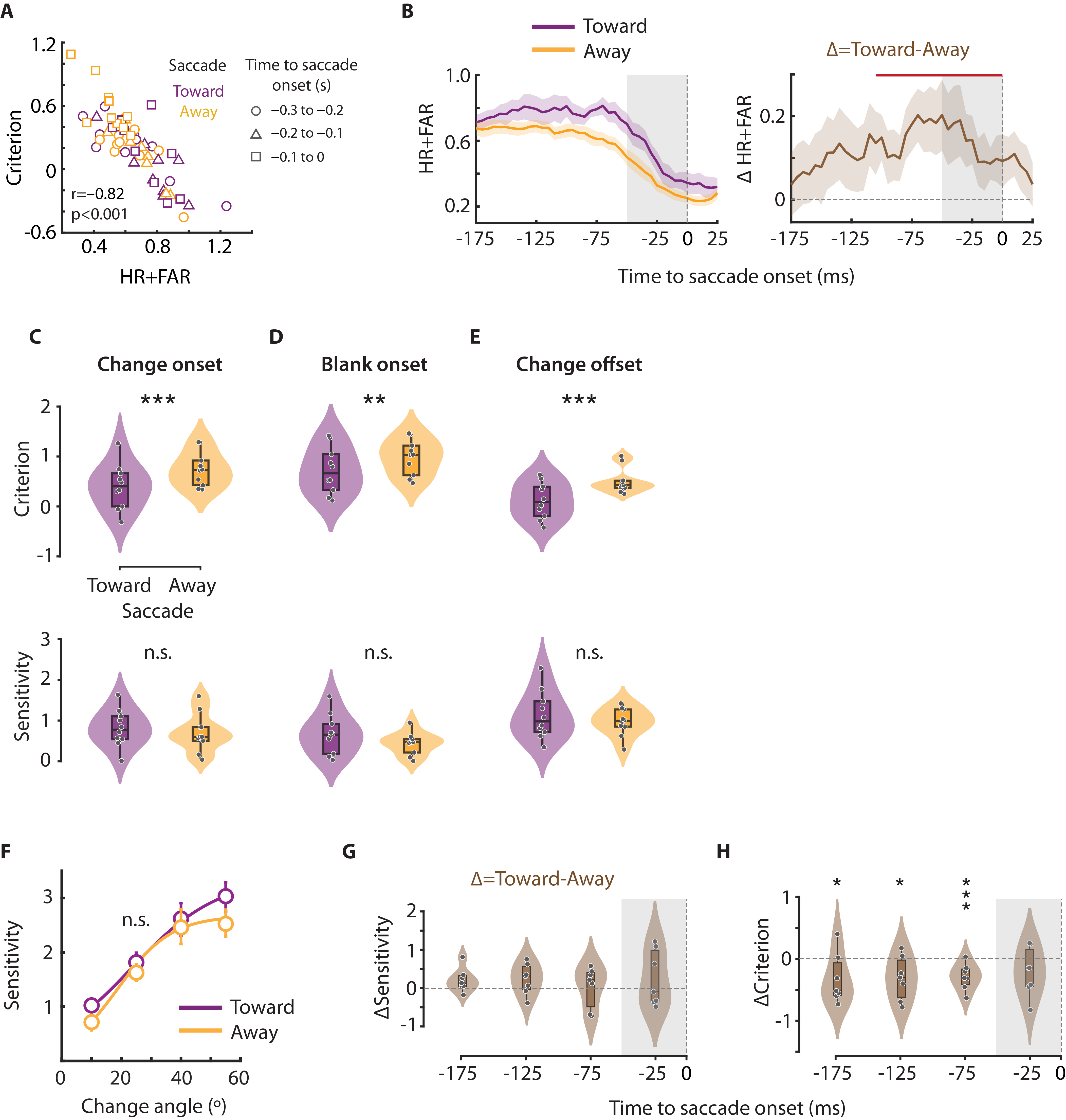

Supplement: S1 Fig — (A) Relationship between estimated criterion (y-axis) plotted against proportion of hits and false alarms (x-axis), at the Saccade Toward (purple) and the Saccade Away (orange) locations. Distinct symbols denote distinct time bins prior to saccade onset. Circle: –300 to –200 ms, triangle: –200 to –100 ms, square: –100 to 0 ms. Each point denotes 1 time bin per participant. (B) (Top) Temporal dynamics of the sum of hit and false alarm rates at the Saccade Toward (purple) and Away (orange) locations locked to saccade onset (dashed vertical line). (Bottom) Presaccadic modulation of hit and false alarm rates locked to saccade onset (Δ = Toward–Away). Solid line: Mean values across participants, shaded error bars: SEM. Shaded gray zone: saccadic suppression epoch. Red horizontal line: cluster of significant differences. Other conventions are the same as in Fig 1E. (C) Criterion (top) and sensitivity (bottom) for orientation change detection reports at the Saccade Toward and Saccade Away locations, for trials with change onset <70 ms before the saccade onset. Other conventions are the same as in Fig 1E. (D) Same as in panel C, but showing criterion and sensitivity for trials with blank onset <70 ms before the saccade onset. (E) Same as in panel C, but showing criterion and sensitivity for trials with change offset <70 ms before the saccade onset. (F) Psychometric function of sensitivity at the Saccade Toward and Saccade Away locations for orientation change detection and localization with multiple change angles (10°, 25°, 40°, and 55°). Error bars: SEM (n = 5 participants). (G) Same as in Fig 1I, but showing temporal dynamics of difference in sensitivity at the Saccade Toward and Away locations for the contrast change detection task. (H) Same as in Fig 1J, but showing temporal dynamics of difference in criterion at the Saccade Toward and Away locations for the contrast change detection task. Data are available at https://dx.doi.org/10.6084/m9.figshare.21792002. (TIF) [file pbio.3002485.s001.tif]

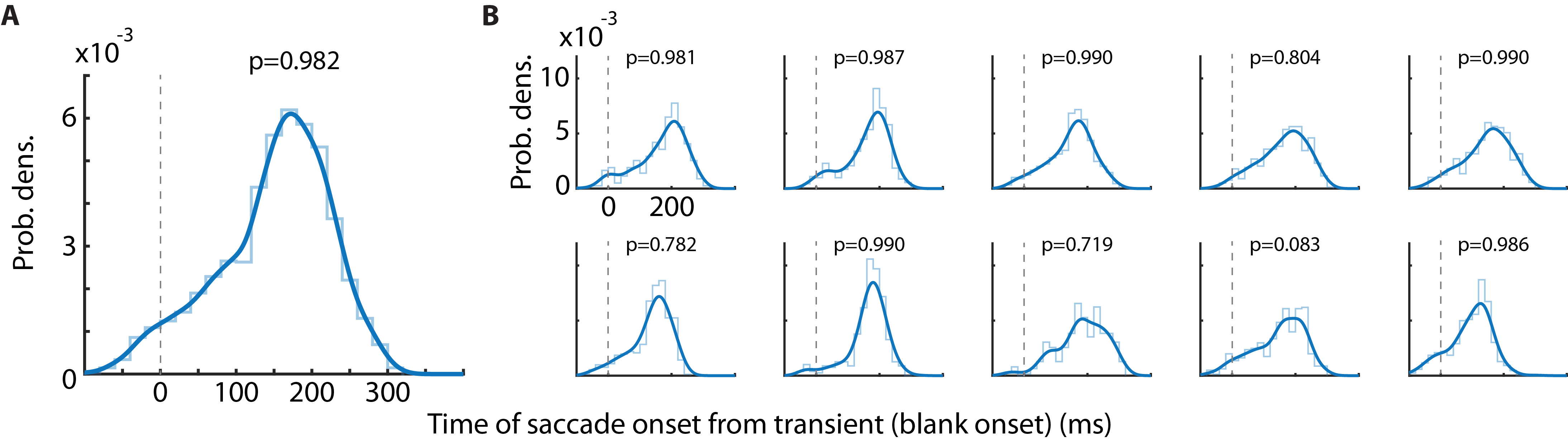

Supplement: S2 Fig — (A) Same as in Fig 1C, but showing the distribution of saccade onset times locked to the time of transient (blank onset), pooled across participants, in the orientation change detection task. Annotation: p-values for Hartigan’s dip test of unimodality. Other conventions are the same as in Fig 1C. (B) Same as in panel A, but showing the distribution of saccade onset times locked to the time of transient for each participant (distinct plots). Data are available at https://dx.doi.org/10.6084/m9.figshare.21792002. (TIF) [file pbio.3002485.s002.tif]

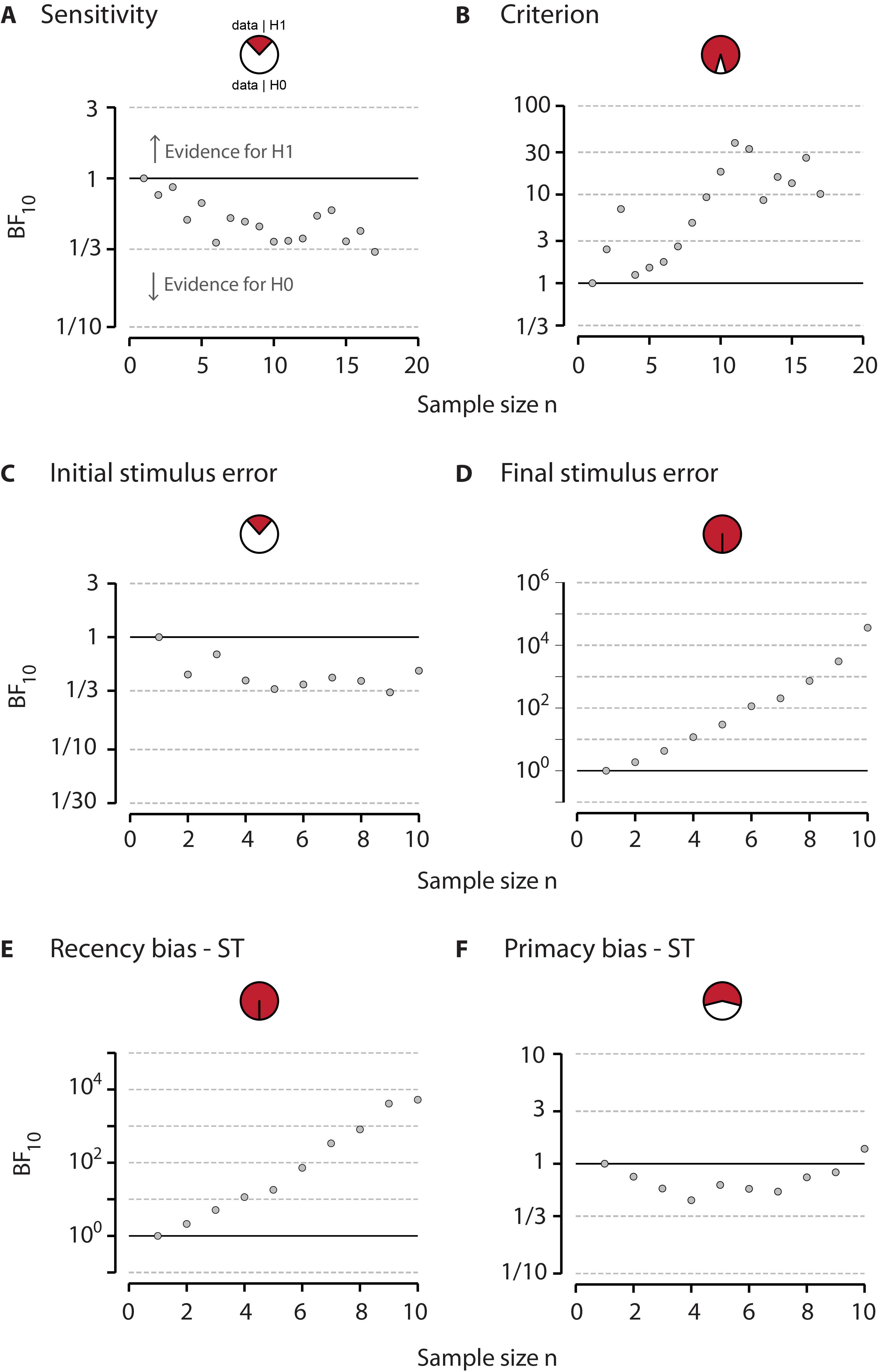

Supplement: S3 Fig — (A) Bayesian Sequential Analysis based on a paired sample t test, as a function of sample size, for d’ difference between the Saccade Toward (ST) and Saccade Away (SA) locations in the change detection tasks. Samples are pooled over the orientation change (n = 10) and contrast change (n = 7) detection experiments to yield a total sample size of n = 17. Here, H0 is the hypothesis that d’ is not different between the ST and SA locations while H1 is the alternative hypothesis that d’ is different across these locations. The x-axis represents the sequential sample sizes (from n = 1 to n = 17 participants), the left y-axis indicates the Bayesian Factor supporting H1 over H0 (BF10), and the right y-axis provides labels for different BF levels. Similarly, BF01 quantifies the evidence supporting H0 over H1. (Inset top-center) Pie chart indicates the data likelihoods under the 2 hypotheses (white: H0 and red: H1). (B) Same as in panel (A) but for criterion difference between the ST and SA locations in the change detection tasks. Other conventions are the same as in panel A. (C) Same as in panel (A) but for a difference in initial stimulus precision between the ST and SA locations, in “double set” trials, in the orientation estimation task (n = 10). (D) Same as in panel (C) but for a difference in final stimulus precision between the ST and SA locations, in “double set” trials, in the orientation estimation task (n = 10). (E) Same as in panel (A) but for recency bias at the ST location in the orientation estimation task (n = 10). (F) Same as in panel (E) but for primacy bias at the ST location in the orientation estimation task (n = 10). (B–F) Other conventions are the same as in panel A. (TIF) [file pbio.3002485.s003.tif]

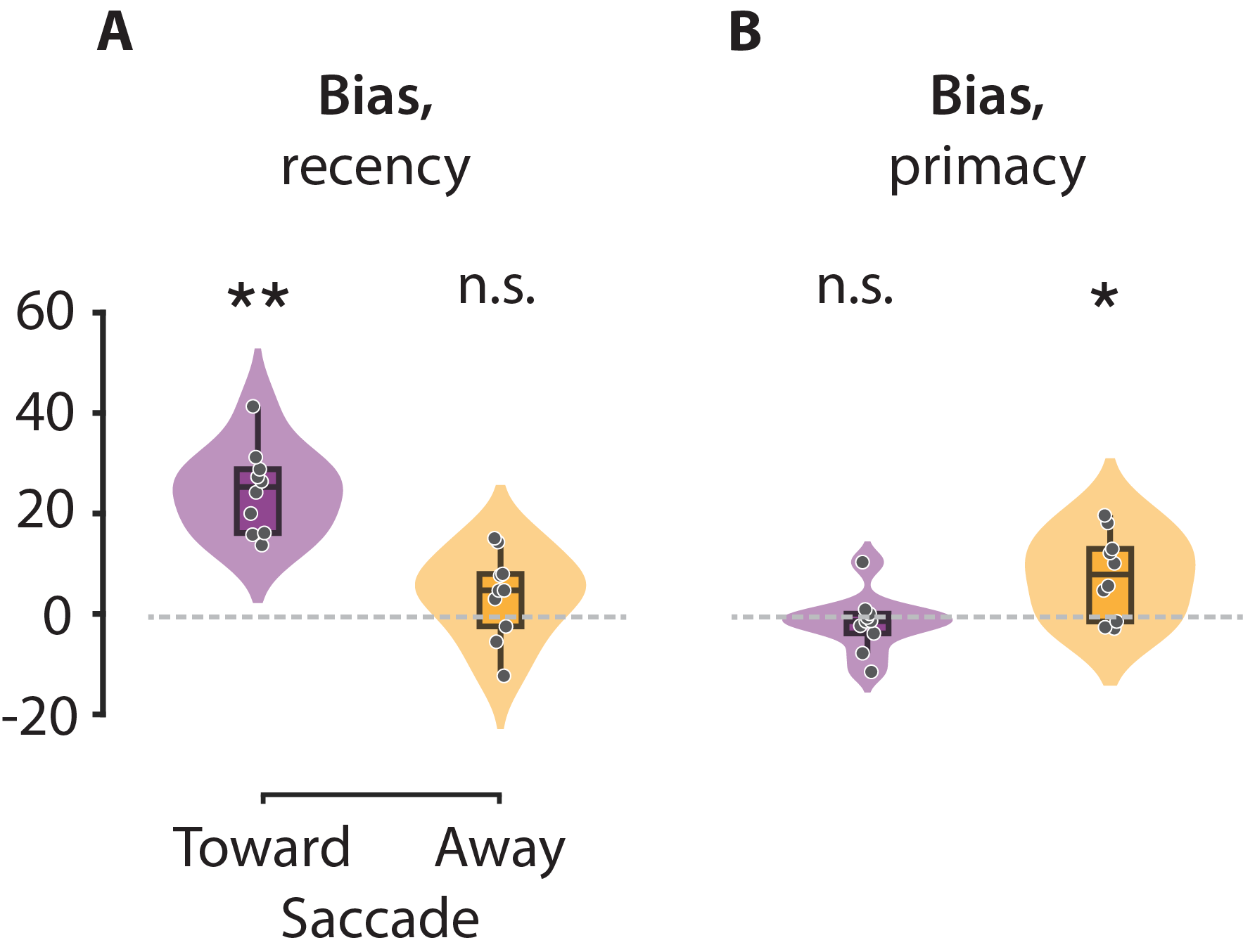

Supplement: S4 Fig — (A) Same as in Fig 3C (main text), but showing recency bias at the Saccade Toward and Saccade Away locations, following exclusion of trials in which the saccade onset occurred <50 ms after final stimulus offset. (B) Same as in panel A, but showing primacy bias at the Saccade Toward and Saccade Away locations. (A, B) Other conventions are the same as in Fig 3C. Data are available at https://dx.doi.org/10.6084/m9.figshare.21792002. (TIF) [file pbio.3002485.s004.tif]

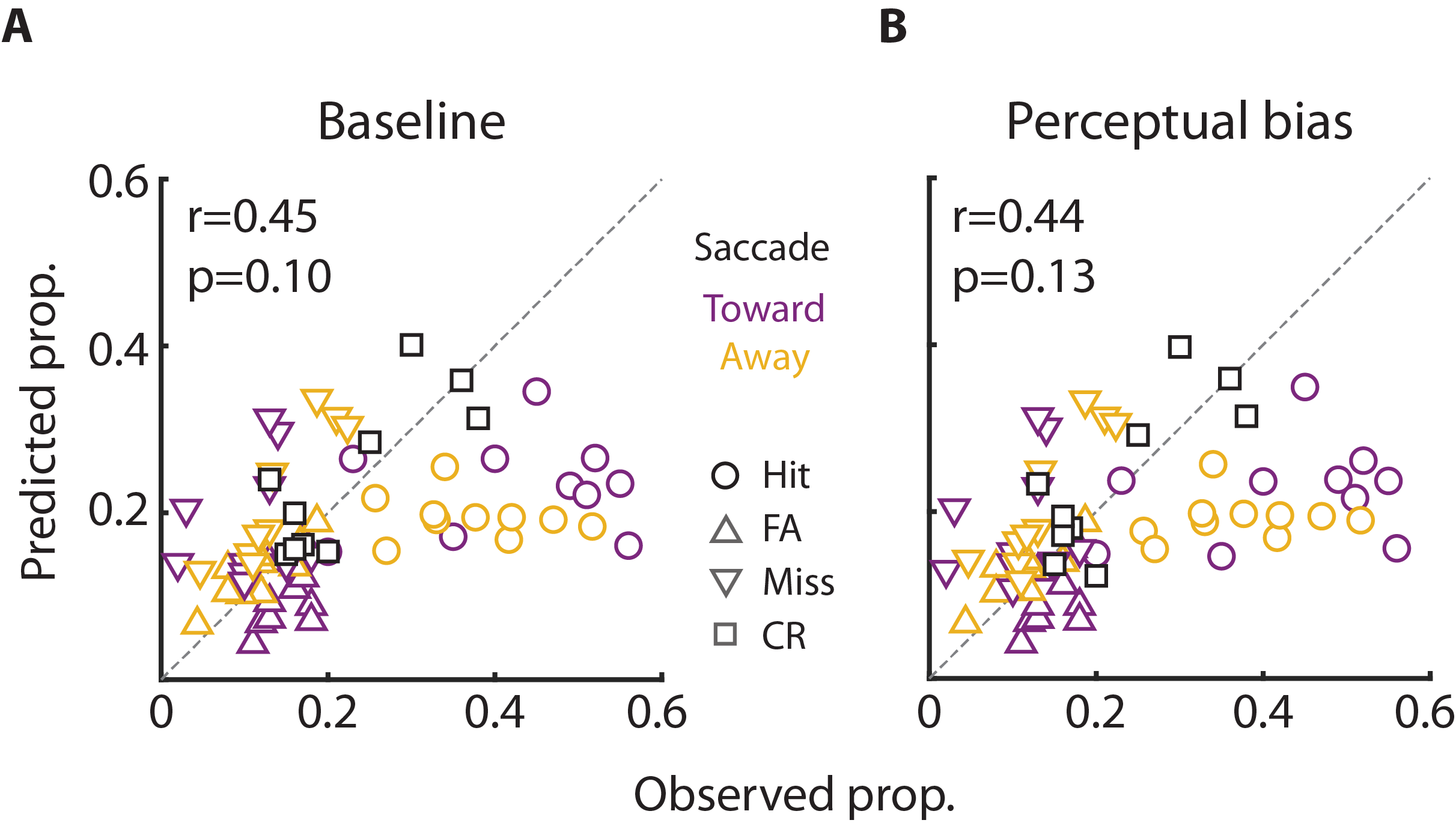

Supplement: S5 Fig — (A) Same as in Fig 5E (main text), but showing response proportions predicted with the baseline model (y-axis) versus observed (true) response proportions (x-axis) in the change detection task. (B) Same as in Fig 5E (main text), but showing response proportions predicted with the perceptual bias model (y-axis) versus observed (true) response proportions (x-axis) in the change detection task. (A, B) Other conventions are the same as in Fig 5E. Data are available at https://dx.doi.org/10.6084/m9.figshare.21792002. (TIF) [file pbio.3002485.s005.tif]
